# Synthesis, Structure, and Reactivity of Hypervalent Iodine Reagents Stabilized by Internal Out-of-Plane Halogen Bonding: *ortho-*Bromo and *ortho-*Chloro-Substituted [Hydroxy(tosyloxy)iodo]arenes and Iodonium Ylides

**DOI:** 10.1021/acs.joc.6c00423

**Published:** 2026-06-22

**Authors:** Niloofar Zarrabi, Grayson Hughes, Brianna Herrmann, Simon Krystosek, Daniel R. Tyson, Yoshi Funk, Gregory T. Rohde, Viktor V. Zhdankin

**Affiliations:** † Department of Chemistry and Biochemistry, 14713 University of Minnesota Duluth, Duluth, Minnesota 558120, United States; ‡ 348905 Marshall School, Duluth, Minnesota 55811, United States

## Abstract

The preparation and reactivity of a series of *ortho*-halogen-substituted (diacetoxyiodo)­arenes, [hydroxy­(tosyloxy)­iodo]­arenes, and aryliodonium ylides are reported. X-ray analysis of 1-[hydroxy­(tosyloxy)­iodo]-2-bromobenzene confirms the presence of halogen bonds in the intramolecular interactions between the *ortho*-halogen atom and the hypervalent iodine center, with a normalized contact (observed contact length divided by the sum of the van der Waals radii) value of 0.91. The *o*-bromo-substituted aryliodonium ylide can be handled and stored at room temperature and can serve as a convenient carbene precursor in a Rh-catalyzed cyclopropanation reaction with styrenes, affording the corresponding cyclopropane products in 59–75% yields.

## Graphical abstract

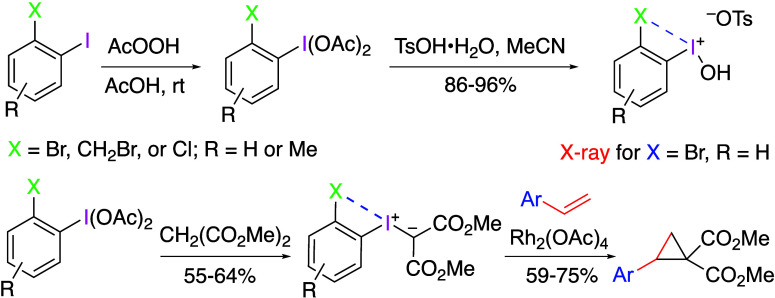

## Introduction

In recent years, the concept of halogen bonding has been widely utilized in the structural chemistry of hypervalent iodine compounds.^1^ According to the IUPAC, a halogen bond (XB) is defined as an “attractive interaction between an electrophilic region associated with a halogen atom in a molecular entity and a nucleophilic region in another, or the same, molecular entity”.^2^ The classical XB involves the electrophilic region (σ-hole) opposite to the covalent bond in monovalent halogen compounds (Figure 1a); however, in modern literature, the concept of XB is commonly used for the structural interpretation of secondary bonding in hypervalent iodine compounds.^1a,1b^ According to DFT-level computational studies, iodonium compounds (R_2_I^+^) possess two σ-holes opposite to the covalent bonds I–C, as shown in Figure 1b.^1f^ Halogen bonding is a well-recognized noncovalent interaction in iodonium salts, widely utilized in areas such as rational supramolecular design^1g–1i^ and organocatalysis.^1j–1l^ Halogen bonding extends to the ylidic compounds as well.^1f,1m^ Furthermore, as more geometric arrangements of electrophilic halogen atoms and nucleophilic donor atoms are observed, the definition of XB has been proposed to expand.^3^ Electron-deficient regions located above the pseudosquare planar coordination environment of hypervalent halogen atoms were observed to interact in an apparent electrostatic manner with nucleophiles and have been coined π-hole features.^4^ These π-hole features may be visualized as a region deficient in electron density between the T-shaped (or *pseudo*-square planar) coordination sphere and the lone pairs in the λ^3^-iodane system.

**Figure 1:**
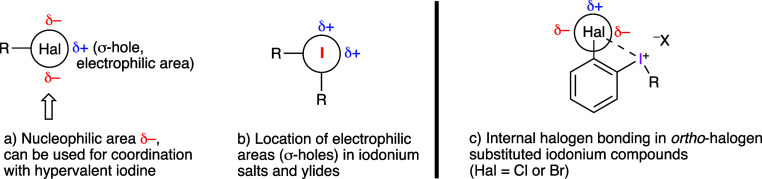
Utilization of the concept of internal halogen bonding for stabilization of *ortho*-halogen-substituted iodonium compounds.

The presence of internal halogen bonding between hypervalent iodine and a halogen atom (e.g., bromine) in the *ortho*-position (Figure 1c) is expected to stabilize the molecule, improve solubility, and modify the reactivity of a hypervalent iodine compound. The ability of a halogen to participate in halogen bonding as an acceptor follows the overall trend: I > Br > Cl > F.^1a^ Despite its obvious simplicity, the concept of stabilization of hypervalent iodine compounds by internal halogen bonding with a neighboring halogen atom was not previously explored in the literature; however, the preparation of several iodine­(III) compounds of this type [e.g., 2-BrC_6_H_4_I­(OAc)_2_ and 2-BrC_6_H_4_I­(OH)­OTs] was previously reported as part of unrelated synthetic studies.^5^ In contrast, numerous derivatives bearing *ortho-*O- or N-coordinating substituents and stabilized by internal I•••O or I•••N coordination have been reported, and some of these pseudocyclic compounds have found wide synthetic application as reagents and catalysts.^6^ It has been demonstrated that pseudocyclic hypervalent compounds have improved thermal stability and higher solubility, often accompanied by improved reactivity patterns.^6^ At the same time, however, the *ortho*-substituted pseudocyclic hypervalent iodine compounds derived from 2-iodophenols, anilines, or carboxylic esters have relatively low stability to strong acids and strong oxidants, which restricts their practical application. The *ortho-*halogen (Br or Cl) substituted hypervalent iodine compounds should have enhanced stability toward strong acids and strong oxidants.

## Results and Discussion

Herein, we report the preparation, structural study, and chemical reactivity of a series of *ortho*-halogen-substituted (diacetoxyiodo)­arenes, [hydroxy­(tosyloxy)­iodo]­arenes, and aryliodonium ylides. In the first step, we prepared *ortho*-bromo, *ortho*-chloro, and *ortho*-bromomethyl-substituted (diacetoxyiodo)­arenes **1**–**5** by the peracetic oxidation of the respective commercially available aryl iodides (Scheme 1). The reactions were performed on a 10 mmol scale, allowing the safe preparation of pure products in 2–4-g quantities with good yields. All products **1**–**5** were isolated as thermally stable, white, microcrystalline solids and identified by NMR and high-resolution ESI spectra. (Diacetoxyiodo)­arenes **1**–**4** were previously reported in the literature.^5^

**Scheme 1:**
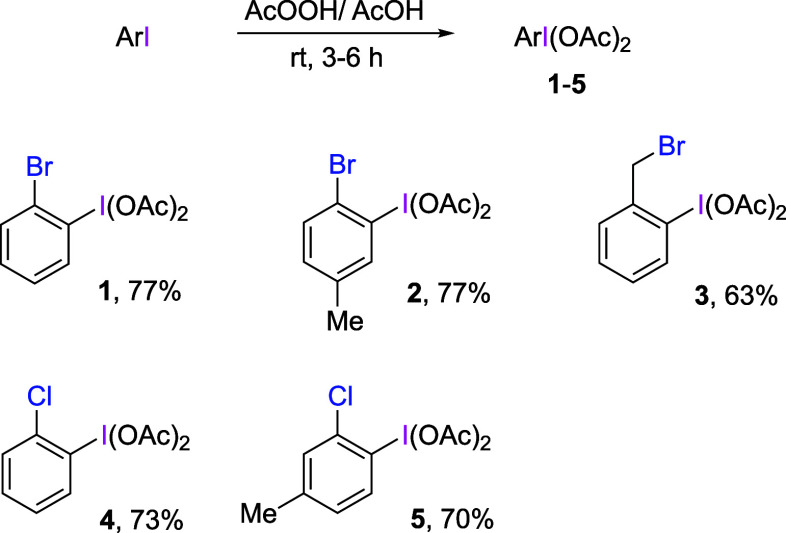
Synthesis of (Diacetoxyiodo)­arenes **1**–**5**

(Diacetoxyiodo)­arenes **1**–**5** were further converted to the corresponding [hydroxy­(tosyloxy)­iodo]­arenes **6**–**10** by reaction with *p*-toluenesulfonic acid monohydrate in acetonitrile at room temperature (Scheme 2). Tosylates **6**–**10**, were obtained as thermally stable, white, microcrystalline solids by cooling the initially formed yellow solution to 0 °C and were identified by NMR, high-resolution ESI mass spectrometry, and X-ray crystallography (for compound **6**).

**Scheme 2:**
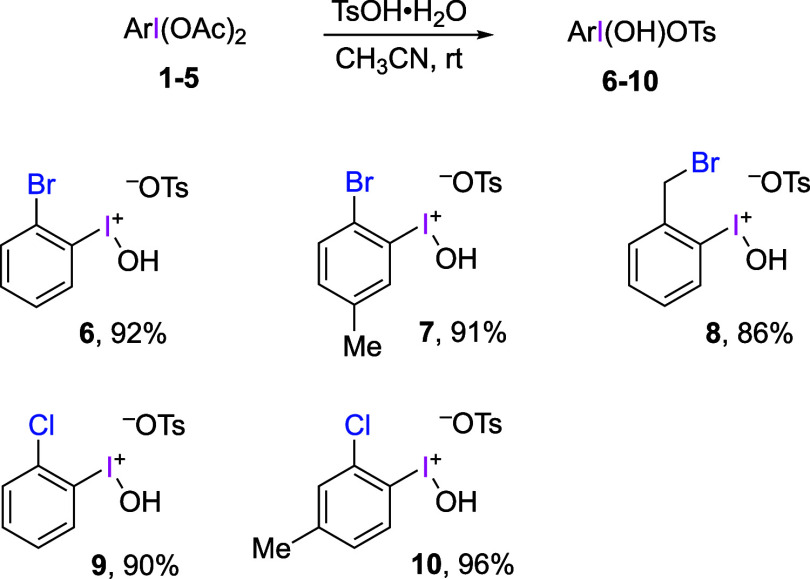
Synthesis of [Hydroxy­(tosyloxy)­iodo]­arenes **6**–**10**

[Hydroxy­(tosyloxy)­iodo]­arene **6** was studied by single-crystal X-ray diffraction and was found as a dimer formed by intermolecular halogen bonding between the iodine atom of one molecule and an oxygen atom of a tosylate group of a second molecule, related by an inversion center (Figure 2). Classic σ-hole halogen bonding is supported by normalized contact (Nc) distances of the oxygen atom to the iodine atom of 0.80 and C1–I1–O3 bond angles of 169(1)°.^7^ Normalized contact (Nc) is defined as the observed contact length divided by the sum of the van der Waals radii.^7^ An Nc value less than 1 provides evidence of a noncovalent interaction between an electrophilic halogen atom and a nucleophilic donor.^7^ The geometry of the first coordination sphere of the iodine atom is assigned as square planar due to a nearly square planar base. A root-mean-square distance from a plane was calculated for the atoms I1–C1–O1–O2–O3 as 0.068 Å, and bond angles close to 180° (Figure 2) support the assignment. A nonstandard close contact between I(1) and Br(1) was observed with an Nc distance of 0.91 (Figure 2). A search of the Crystallographic Structural Database (CSD) version 6.01 for motifs similar to 2-bromoiodobenzene starting material yielded five structures (reference numbers CIVWAJ, DADQAE, KATZOX, MOKDOJ, HIGTIE).^8^ The average I–Br distance was 3.51 Å, with an average Nc of 0.92. No noticeable difference was observed, however, a dihedral angle of 63(1)° between Br1–C1–I1–O1 was observed and is consistent with the Br atom acting as a nucleophile pointing at a π-hole of the iodine­(III) atom. Two similar ortho-substituted structures were found in a search of the CSD version 6.01 (references MUKJEJ and CHNPIH10).^9^ The related structures contain three out-of-plane halogen bonding pairs (one I–Cl and two I–F) and were observed with an average Nc of 0.92 and a dihedral angle of 69°. The I–Br Nc ratio of 0.91 and the dihedral angle close to 65° between the lone pairs and the pseudosquare planar coordination environment of the λ^3^–iodane system support the assignment of an out-of-plane (π-hole) halogen bond.

**Figure 2:**
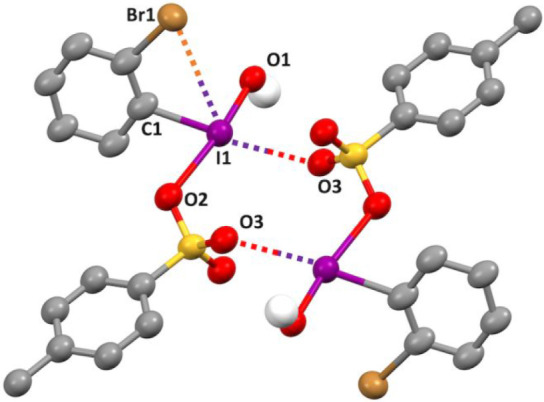
Perspective view of a dimer of compound **6**. Thermal ellipsoids drawn to the 50% probability level, and nonoxygen hydrogen atoms were removed for clarity. Atom colors: C – gray, O – red, I – purple, Br – brown, S – yellow. Selected bond and contact distances in Å and Normalized Contact distances [Nc]: I1–C1 2.19(3), I1–O1 1.97(3), I1–O2 2.50(3), I1–O3 2.81(2) [0.80], I1–Br1 3.483(4) [0.91]. Selected bond angles (°): O1–I1–O2 173.5(9), C1–I1–O3 169(1). Dihedral bond angles (°): Br1–C1–I1–O1 63(1), CCDC 2525585.

X-ray analysis of structure **6** confirms the presence of a halogen bond in the intramolecular interactions between the *ortho-*halogen atoms and the hypervalent iodine center, with a normalized contact distance of 0.91. It is known that additional coordination of iodine in hypervalent iodine compounds improves their stability and solubility, which is particularly important in hypervalent iodine reagents.^6b^ Compounds **6** and **9** are stable for storage at room temperature and melt at 135–140 °C without decomposition, which is similar to the unsubstituted [hydroxy­(tosyloxy)­iodo]­benzene, PhI­(OH)­OTs.

To gain further insight into the stabilizing effect of the *ortho-*halogen atoms, we have synthesized malonate-derived *ortho*-halogen-substituted aryliodonium ylides. It is known from the literature that the unsubstituted phenyliodonium ylide derived from dimethyl malonate has low thermal stability at room temperature and decomposes at about 100 °C.^10^

The target malonate-derived *ortho*-halogen aryliodonium ylides **11**–**16** were prepared in moderate yields by the reaction of (diacetoxyiodo)­arenes **1**–**5** with commercially available methyl, ethyl, or isopropyl malonate esters (Scheme 3) using the previously reported common synthetic procedures for iodonium ylides (see Supporting Information for General Procedures A and B).^10,11^

**Scheme 3:**
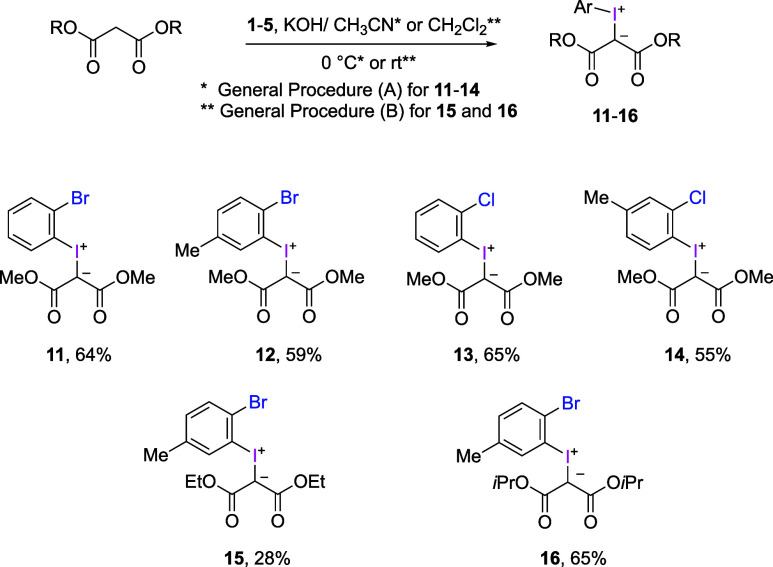
Synthesis of *ortho*-Halogen-Substituted Aryliodonium Ylides **11**–**16**

All products **11**–**16** were isolated as relatively stable, off-white, microcrystalline solids and identified by NMR and high-resolution ESI spectra. Iodonium ylide **11** has a melting point of 132–134 °C without noticeable decomposition, which is indicative of higher thermal stability compared to the unsubstituted phenyliodonium ylide, which has a melting point of 100−104 °C (softens to gum^10a^). Aryliodonium ylides **11**–**16** can be stored indefinitely in a refrigerator at 4 °C. According to literature data, the unsubstituted phenyliodonium ylide, PhIC­(CO_2_CH_3_)_2_, “··· degrades slowly at room temperature (over 2 weeks) ... but it can be stored at −20 °C for months without significant degradation”.^10a^ We have tested the stability of ylides **11**, **12**, and **13** in the solid state at room temperature by keeping the samples on the lab bench for 4 weeks. During this time, their decomposition was monitored by recording ^1^H NMR spectra in CDCl_3_ (see Supporting Information). The NMR results showed good stability for ylide **11**, but some degradation was observed for ylide **13**, and significant degradation was noted for ylide **12,** leading to a color change from white to yellow.

Phenyliodonium ylides have found application in organic synthesis as efficient carbene precursors, serving as an alternative to diazo compounds but without major drawbacks such as explosiveness and toxicity.^12^ Phenyliodonium bis­(methoxycarbonyl)­methanide, PhIC­(CO_2_CH_3_)_2_, the most common iodonium ylide, has found synthetic applications in the cyclopropanation of alkenes, C–H insertion reactions, and asymmetric synthesis.^12b^ We have tested the reactivity of iodonium ylide **11** in a Rh-catalyzed cyclopropanation reaction with styrenes under previously optimized,^10,11^ standard reaction conditions (Scheme 4). As expected, these reactions afforded the corresponding cyclopropane products **17**–**20** in good yields, similar to the reactions of the unsubstituted phenyliodonium ylide PhIC­(CO_2_CH_3_)_2_ ^10^ and comparable to the reactions of the *ortho*-methoxy-substituted iodonium ylide;^11^ however, in contrast to the previously known reagents, iodonium ylide **11** is a stable compound that can be handled and stored at room temperature.

**Scheme 4:**
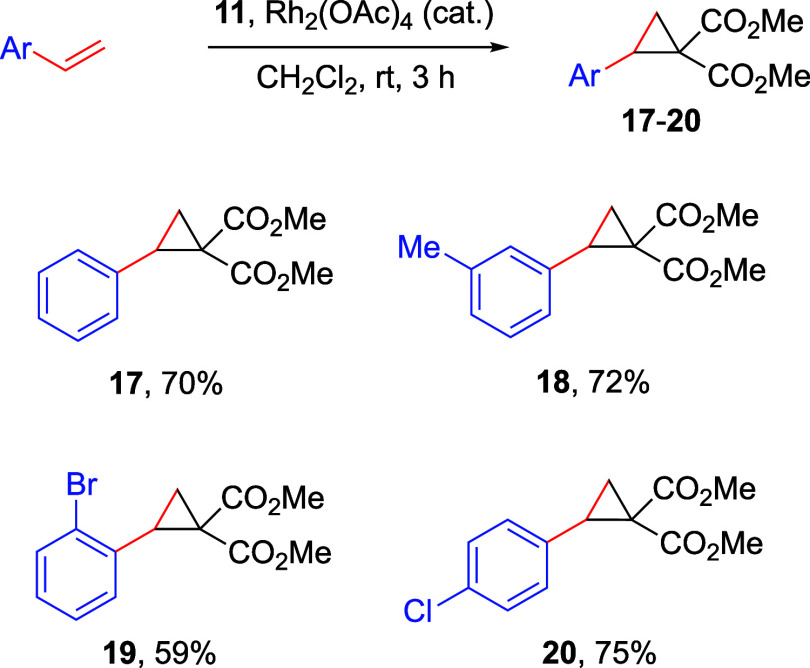
Catalytic Cyclopropanation of Styrenes Using Aryliodonium Ylide **11**

## Conclusion

In summary, we have reported the preparation, X-ray structure, and reactions of a series of *ortho*-halogen-substituted (diacetoxyiodo)­arenes, [hydroxy­(tosyloxy)­iodo]­arenes, and aryliodonium ylides. X-ray analysis of the tosylate **6** confirms the presence of halogen bonds in the intramolecular interactions between the *ortho*-halogen atom and the hypervalent iodine center, with a normalized contact distance of 0.91. Compared to the common unsubstituted phenyliodonium ylide PhIC­(CO_2_CH_3_)_2_, the *o*-bromo-substituted aryliodonium ylide **11** is a stable compound that can be handled and stored at room temperature. The reactivity of iodonium ylide **11** in the synthetically important Rh-catalyzed cyclopropanation reaction of alkenes is similar to that of the unsubstituted phenyliodonium ylide and comparable to the reactions of the *o*-methoxy-substituted iodonium ylide.

## Supplementary Material



## Data Availability

The data underlying this study are available in the published article and its Supporting Information.

## Notes

Detailed experimental procedures, analytical data, NMR spectra of the synthesized compounds, and X-ray data for compound **6** (PDF)
