# Supplementary material for: Synthesis, Structure, and Reactivity of Hypervalent Iodine Reagents Stabilized by Internal Out-of-Plane Halogen Bonding: ortho-Bromo and ortho-Chloro-Substituted [Hydroxy(tosyloxy)iodo]arenes and Iodonium Ylides
Source: J Org Chem. 2026 Jun 22;91(26):8820–4. doi: 10.1021/acs.joc.6c00423 (PMC13339633; doi:10.1021/acs.joc.6c00423)

Supporting Information

Synthesis, Structure, and Reactivity of Hypervalent Iodine Reagents Stabilized by Internal Out-of-Plane Halogen Bonding: *ortho*-Bromo and *ortho*-Chloro Substituted [Hydroxy(tosyloxy)iodo]arenes and Iodonium Ylides

Niloofar Zarrabi,[a] Grayson Hughes,[a] Brianna Herrmann,[a] Simon Krystosek,[a] Daniel R. Tyson,[a] Yoshi Funk,[a] Gregory T. Rohde,*[b] and Viktor V. Zhdankin*[a]

[a]*Department of Chemistry and Biochemistry, University of Minnesota Duluth, Duluth, Minnesota, 558120, USA*

[b]*Marshall School, Duluth, Minnesota 55811, USA*

**Corresponding Author:* vzhdanki@d.umn.edu

Table of Contents

		pp
1.	General experimental remarks	S2
2.	Preparation of (diacetoxyiodo)arenes **1-5**	S2
3.	Preparation of [(hydroxy)tosyloxy]iodoarenes **6-10**	S5
4.	X-ray crystal data for compounds **6** and **9**	S9
5.	Preparation of iodonium ylides **11-16**	S10
6.	Stability of iodonium ylides	S14
7.	Cyclopropanation of styrenes with iodonium ylide **11**	S18
8.	References	S21
6.	NMR Spectra of products	S23

1. General experimental remarks

The reactions requiring an inert atmosphere were conducted under argon atmosphere using standard Schlenk line techniques and oven-dried glassware. The chemicals and solvents utilized in this study were purchased from Sigma-Aldrich, Fisher, Alfa-Asear, or AmBeed and were used as received. Anhydrous solvents were obtained by distillation over calcium hydride before use unless otherwise stated. Chromatographic materials were purchased from SiliCycle or Sigma-Aldrich. NMR spectra were recorded using a Bruker 400 MHz NMR spectrophotometer (^{1}H NMR and ^{13}C NMR). Chemical shifts are reported in parts per million (ppm). Coupling constants were quoted in Hz (*J*). ^{1}H NMR spectroscopy splitting patterns were designated as singlet (s), doublet (d), or triplet (t). Splitting patterns that could not be interpreted or easily visualized were designated as multiplet (m). ESI mass spectra were recorded on a Bruker MicroTOF-III mass spectrometer using anhydrous acetonitrile. Meting points were determined in an open capillary tube with a Mel-temp II melting point apparatus. X-ray crystal analysis was performed by Rigaku RAPID II XRD Image Plate using graphite-monochromated Cu Kα radiation (λ = 1.54187 Å) at 125 K.

2. Preparation of (diacetoxyiodo)arenes 1-5[1]

General procedure:

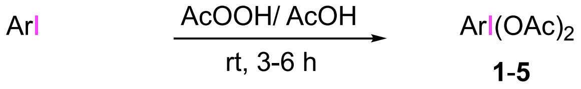

Caution! *Peracetic acid is a strong oxidizing and corrosive agent (GHS Category 1, Codes H242 and H290). It may cause severe skin burns and irreversible eye damage (GHS Category 1, Codes H312, 314, and H318). Always wear chemical safety goggles, protective gloves, and respirators when handling peracetic acid.*

(Diacetoxyiodo)arenes **1-5** were prepared by the oxidation of the respective commercially available aryliodides (10.0 mmol, 1.0 equiv.) using 32% commercial solution of peracetic acid in acetic acid (5.7 mL, 27.0 mmol, 2.7 equiv.). The peracetic acid solution was added dropwise to the round bottom flask containing the aryliodides over 30 minutes at room temperature. The reaction mixtures were stirred for an additional 3-6 hours. After removing solvents under reduced pressure, the solid was washed with 3 x 15 mL water, followed by 3 x 15 mL hexane and 3 x 15 mL diethyl ether, and dried under vacuum.

*1-(Diacetoxyiodo)-2-bromobenzene **1***

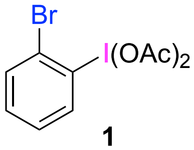

1

1-(Diacetoxyiodo)-2-bromobenzene **1** was prepared by following the general procedure from 1-bromo-2-iodobenzene (1.3 mL, 10.0 mmol, 1.0 equiv.) and peracetic acid in acetic acid solution (5.7 mL, 27.0 mmol, 2.7 equiv.). The reaction mixture was stirred for a total of 4 hours. The product was obtained as a white solid (3.10 g, 77%): mp 143-144 °C; ^{1}H NMR (400 MHz, $CDCl_3$): δ 8.26 (dd, *J* = 7.9, 1.4 Hz, 1H), 7.91 (dd, *J* = 7.9, 1.2 Hz, 1H), 7.51 (td, *J* = 7.5, 1.4 Hz, 1H), 7.42 (td, *J* = 7.8, 1.4 Hz, 1H), 2.03 (s, 6H); HRMS (ESI) *m/z*: $[M + Na]^+$ Calcd for $C_{10}H_{10}O_4NaBrI$ 422.8700; Found 422.8660.

Note: Product **1** was previously reported in the literature with mp 89–91 °C;[2] however, HRMS was not provided in this paper. Our procedure resulted in a better yield of pure product **1** with mp 143-144 °C (without decomposition).

*1-(Diacetoxyiodo)-2-bromo-5-methylbenzene **2***

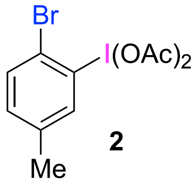

2

Preparation of product **2** was carried out by dissolving 1-bromo-2-iodo-4-methylbenzene (3.0 g, 10.0 mmol, 1.0 equiv.) in a minimum amount of dichloromethane, followed by the general procedure of adding peracetic acid in acetic acid solution (5.7 mL, 27.0 mmol, 2.7 equiv.). The reaction mixture was stirred for a total of 5 hours. The product was obtained as a white solid (3.20g, 77%): mp 153-154 ºC; ^{1}H NMR (400 MHz, $CDCl_3$): δ 8.08- 8.07 (m, 1H), 7.77 (d, J = 8.2 Hz, 1H), 7.31(m, 1H), 2.41 (s, 3H), 2.04 (s, 6H); HRMS (ESI) *m/z*: $[M + Na]^+$ Calcd for $C_{11}H_{12}O_4NaBrI$ 436.8856; Found 436.8831.

Note: Preparation of product **2** in 72% by the perborate oxidation of 1-bromo-2-iodo-4-methylbenzene was previously reported in the literature.[3] However, the melting point and HRMS was not provided in this paper.[3] Our NMR data were consistent with the previously reported spectra.

*1-(Diacetoxyiodo)-2-bromomethylbenzene **3***

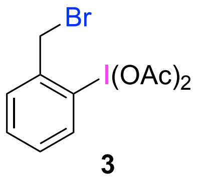

3

Preparation of product **3** was carried out by dissolving 1-(bromomethyl)-2-iodobenzene (3.0 g, 10.0 mmol, 1.0 equiv.) in a minimum amount of dichloromethane, followed by the general procedure of adding peracetic acid in acetic acid solution (5.7 mL, 27.0 mmol, 2.7 equiv.). The reaction mixture was stirred for an additional 4 hours. The product was obtained as a white solid (2.60 g, 63%): mp 110–111 ºC; ^{1}H NMR (400 MHz, $CDCl_3$): 8.26 (d, J = 8.0 Hz, 1H), 7.77 (dd, *J* = 7.8, 1.4 Hz, 1H), 7.65 (td, *J* = 7.6, 0.9 Hz, 1H), 7.41 (td, *J* =7.8, 1.5 Hz, 1H), 4.79 (s, 2H), 2.01 (s, 6H); HRMS (ESI) *m/z*: $[M + Na]^+$ Calcd for $C_{11}H_{12}O_4NaBrI$ 436.8856; Found 436.8834.

Note: Product **3** was previously reported in the literature with mp 113–115 ºC;[4] however, HRMS was not provided in this paper.

*1-(Diacetoxyiodo)-2-chlorobenzene **4***

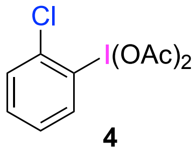

4

Product **4** was prepared by following the general procedure from 1-chloro-2-iodobenzene (1.2 ml, 10.0 mmol, 1.0 equiv.) and peracetic acid in acetic acid solution (5.7 mL, 27.0 mmol, 2.7 equiv.). The reaction mixture was stirred for a total of 6 hours. The product was obtained as a white solid (2.60 g, 73%): mp 146-148 ºC; ^{1}H NMR (400 MHz, $CDCl_3$): δ 8.27 (dd, J = 8.1, 1.2 Hz, 1H), 7.76 (dd, J = 8.0, 1.2 Hz, 1H), 7.60 (td, J = 8.0, 1.3 Hz, 1H), 7.38 (td, J = 7.9, 1.2 Hz, 1H), 2.03 (s, 6H); HRMS (ESI) *m/z*: $[M + Na]^+$ Calcd for $C_{10}H_{10}O_4NaClI$ 378.9205; Found 378.9171.

Note: Product **4** was previously reported in the literature as a white solid with mp 180-197 ºC (sublimated).[1] Our NMR data were consistent with the previously reported spectra.

1-(Diacetoxyiodo)-2-chloro-4-methylbenzene ***5***

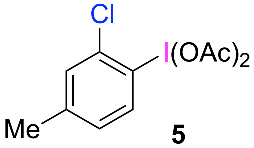

5

Product **5** was prepared by following the general procedure from 3-chloro-4-iodotoluene (1.4 mL, 10.0 mmol, 1.0 equiv.) and peracetic solution in acetic acid (5.7 mL, 27.0 mmol, 2.7 equiv.). The reaction mixtures were stirred for an additional 6 hours. The product was obtained as a white solid (2.6 g, 70%): mp 156-157 ºC; ^{1}H NMR (400 MHz, $CDCl_3$): δ 8.12 (d, J = 8.1 Hz, 1H), 7.55 (s, 1H), 7.16 (d, J = 8,1 Hz, 1H), 2.46 (s, 3H), 2.01 (s, 6H); $^{13}C\{^{1}H\}$ NMR (100 MHz, $CDCl_3$): δ 176.8, 145.2, 138.2, 136.9, 130.4, 129.8, 121.8, 21.4, 20.3; HRMS (ESI) *m/z*: $[M + Na]^+$ Calcd for $C_{11}H_{12}O_4NaClI$ 392.9362; Found 392.9379.

3. Preparation of [hydroxy(tosyloxy)iodo]arenes 6-10[5]

General procedure:

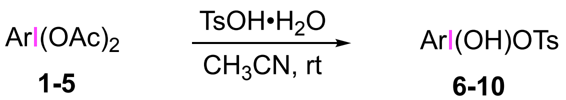

[Hydroxy(tosyloxy)iodo]arenes **6-10** were prepared by adding the solution of *p*-toluenesulfonic acid monohydrate (4.0 mmol, 2.0 equiv.) in 6 mL of acetonitrile into a solution of a (diacetoxyiodo)arene (2.0 mmol, 1.0 equiv.) in 8 mL of acetonitrile at room temperature. The reaction mixture immediately turned into a clear yellow solution. After placing the yellow solution in an ice bath, the product appears as a white solid or crystal within a few hours. It was filtered and washed with 3 x 15 mL acetone and 3 x 15 mL diethyl ether. The product was dried under vacuum.

1-[Hydroxy(tosyloxy)iodo]-2-bromobenzene **6**

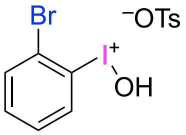

6

Product **6** was prepared by following the general procedure from the reaction of compound **1** (802 mg, 2.0 mmol, 1.0 equiv.) and *p*-toluenesulfonic acid monohydrate (761 mg, 4.0 mmol, 2.0 equiv.). The product was obtained as white crystals (867 mg, 92%): mp 138-140 ºC; ^{1}H NMR (400 MHz, $CDCl_3$): δ 8.29 (d, *J* = 7.8, 1H), 7.87 (d, *J* = 7.7 Hz, 1H), 7.60 (d, *J* = 8.1 Hz, 2H), 7.54-7.50 (m, 1H), 7.40 (t, *J* = 7.6 Hz, 1H), 7.15 (d, *J* = 8.1Hz, 2H), 2.36 (s, 3H); HRMS (ESI) *m/z*: $[M\ -OTs]^+$ Calcd for C_6H_5OBrI 298.8563; Found 298.8546.

Note: Compound **6** was previously reported.[2a,6] In this paper, DMSO was used for NMR spectroscopy; in our work, $CDCl_3$ and 2 drops of CD_3OD were used. Reported mp 137-138 ºC

1-[Hydroxy(tosyloxy)iodo]-2-bromo-5-methylbenzene 7

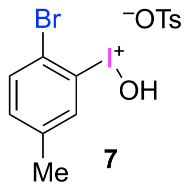

7

Product **7** was prepared by following the general procedure from the reaction of compound **2** (830 mg, 2.0 mmol, 1.0 equiv.) and *p*-toluenesulfonic acid monohydrate (761 mg, 4.0 mmol, 2.0 equiv.). The product was obtained as white crystals (883 mg, 91%): mp 137-139 ºC; ^{1}H NMR (400 MHz, $CDCl_3$): δ 8.07 (s, 1H), 7.73 (d, *J* = 8.2 Hz, 1H), 7.62 (d, *J* = 8.2 Hz, 2H), 7.32 (s, 1H), 7.17(d, *J* = 8.1 Hz, 2H), 2.37(s, 3H), 2.36 (s, 3H); ^{13}C{^{1}H} NMR (100 MHz, $CDCl_3$): δ 141.2, 140.6, 139.7, 139.6, 135.7, 132.7, 128.9, 128.4, 126.0, 124.0, 21.3, 20.6; HRMS (ESI) *m/z*: [M -OTs]$^+$ Calcd for C_7H_7OBrI 312.8720; Found 312.8741.

Note: $CDCl_3$ and 2 drops of CD_3OD used for NMR spectroscopy.

*1-[Hydroxy(tosyloxy)iodo]-2-bromomethylbenzene **8***

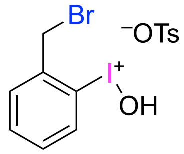

8

Product **8** was prepared by following the general procedure from the reaction of compound **3** (830 mg, 2.0 mmol, 1.0 equiv.) and *p*-toluenesulfonic acid monohydrate (761 mg, 4.0 mmol, 2.0 equiv.). The product was obtained as white crystals (834 mg, 86%): mp 77-79ºC; ^{1}H NMR (400 MHz, $CDCl_3$): δ 8.22 (d, *J* = 8.0 Hz, 1H), 7.68-7.62 (m, 2H), 7.54-7.52 (m, 2H), 7.40 (td, *J* = 8.0, 2.1 Hz, 1H), 7.13(d, *J* = 7.8 Hz, 2H), 4.73 (s, 2H), 2.34 (s, 3H); ^{13}C{^{1}H} NMR (100 MHz, $CDCl_3$): δ 141.5, 140.1, 139.4, 137.9, 134.1, 131.8, 131.2, 129.0, 126.0, 33.6, 21.3 ; HRMS (ESI) *m/z*: [M -OTs]$^+$ Calcd for C_7H_7OBrI 312.8720; Found 312.8714.

Note: $CDCl_3$ and 2 drops of CD_3OD used for NMR spectroscopy.

1-[Hydroxy(tosyloxy)iodo]-2-chlorobenzene ***9***

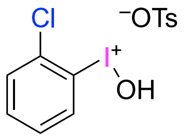

9

Product **9** was prepared by following the general procedure from reaction of compound **4** (713 mg, 2.0 mmol, 1.0 equiv.) and *p*-toluenesulfonic acid monohydrate (761 mg, 4.0 mmol, 2.0 equiv.). The product was obtained as white crystals (769 mg, 90%): mp 136-138 ºC; ^{1}H NMR (400 MHz, $CDCl_3$): δ 8.29 (d, *J* = 8.0, 1H), 7.71 (d, *J* = 7.9 Hz, 1H), 7.62-7.54 (m, 3H), 7.36 (t, *J* = 7.7 Hz, 1H), 7.15 (d, *J* = 7.6 Hz, 2H), 2.36 (s, 3H); HRMS (ESI) *m/z*: [M -OTs]$^+$ Calcd for C_6H_5OClI 254.9069; Found 254.9090.

Note: Compound **9** was previously reported;[6] however, HRMS and melting point were not reported. In the previously reported data, DMSO-d_6 was used for NMR spectroscopy. In our work, $CDCl_3$ and 2 drops of CD_3OD used for NMR spectroscopy.

1-[Hydroxy(tosyloxy)iodo]-2-chloro-4-methylbenzene ***10***

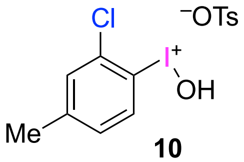

10

Product **10** was prepared by following the general procedure from reaction of compound **5** (741 mg, 2.0 mmol, 1.0 equiv.) and *p*-toluenesulfonic acid monohydrate (761 mg, 4.0 mmol, 2.0 equiv.). The product was obtained as a white solid (846 mg, 96%): mp 134-136 ºC; ^{1}H NMR (400 MHz, $CDCl_3$): δ 8.12 (d, *J* = 8.2, 1H), 7.54 (d, *J* = 8.2 Hz, 2H), 7.46 (s, 1H), 7.12 -7.09 (m, 3H), 2.42 (s, 3H), 2.32 (s, 3H); $^{13}C\{^{1}H\}$ NMR (100 MHz, $CDCl_3$): δ 146.9, 141.1, 139.8, 138.9, 137.2, 130.3, 130.0, 128.9, 125.9, 122.2, 21.4, 21.3; HRMS (ESI) *m/z*: [M -OTs]$^+$ Calcd for C_7H_7OClI 268.9225; Found 268.9202.

Note: $CDCl_3$ and 2 drops of CD_3OD used for NMR spectroscopy.

4. X-ray crystal data for compound 6

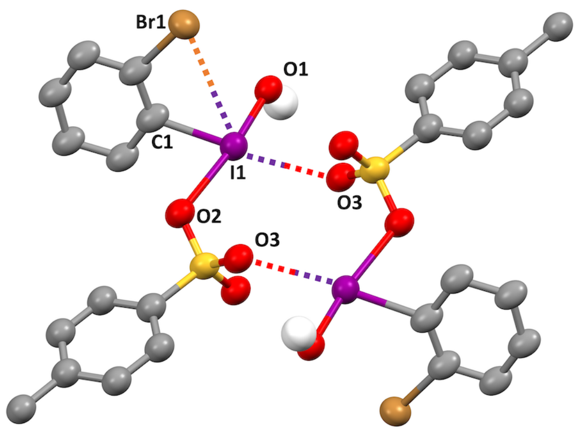

Figure 1S: Structure of a dimer of compound **6**. Thermal ellipsoids drawn to the 50% probability level and hydrogen atoms were removed for clarity. Atoms colors: C-gray, O-red, I-purple, Br-brown, S-yellow.

Single crystals of compound **6** suitable for X-ray crystallographic analysis were obtained by slow evaporation from CH_3CN solution. X-ray diffraction data for **6** were collected on Rigaku RAPID II Image Plate system using graphite-monochromated CuKα radiation (λ = 1.54187 Å) at 123 K. The structure was solved by the Sir 2004 and refined by full-matrix least-squares refinement on F^2 using SHELXL-2014/7. Crystal data for **3a** $C_{13}H_{12}BrIO_4S$: MW 471.11, monoclinic, space group P2/1n, a = 6.7838(3), b = 11.9247(5), c = 18.7396(13) Å, α = 90, β = 90.271(6), γ = 90 o, V =1515.92(14) $Å_3$, Z = 4, 7998 reflections measured, 2595 unique, 1156 I>2σ; final R_1 = 0.1369, S = 0.8328. CCDC number 2525585 contains the supplementary crystallographic data for this paper. These data are provided free of charge by the joint Cambridge Crystallographic Data Centre and Fachinformationszentrum Karlsruhe

5. Preparation of iodonium ylides 11-16

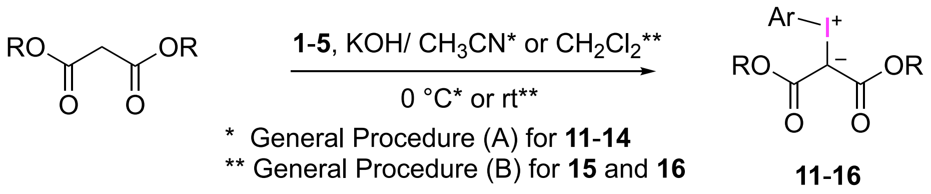

*General procedure (A) for ylides **11-14**:* [7,9]

To a 25 mL flask containing KOH (337 mg, 6.0 mmol, 6.0 equiv.) and dimethyl malonate (116 µL, 1.0 mmol, 1.0 equiv.) under argon atmosphere, 5 ml dry CH_3CN was added. The heterogeneous mixture was placed in an ice/water bath to reach 0 ºC. Then it was stirred vigorously for 5 minutes to form a milky white suspension, followed by the addition of the (diacetoxyiodo)arene (1.1 mmol, 1.1 equiv.) in one portion. The reaction mixture was stirred at 0 ºC, which gradually formed a thick creamy mixture. After 2 hours, 2 mL of cold water was added, and the mixture was stirred for 1 minute. A fluffy white suspension formed in a yellow solution. It was filtered and washed with 3 x 5 mL water and 3 x 5 mL diethyl ether. It was dried under vacuum.

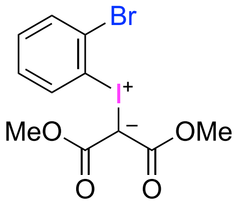

11

Product **11** was prepared by following the general procedure (A) from the reaction of compound **1** (441 mg, 1.1 mmol, 1.1 equiv.) and dimethyl malonate (116 µL, 1.0 mmol, 1.0 equiv.) together with KF (337 mg, 6.0 mmol, 6.0 equiv.) in 5 mL CH_3CN. The product was obtained as a white solid (291 mg, 64%): mp 132-134 ºC; ^{1}H NMR (400 MHz, $CDCl_3$): δ 7.61 (dd, J = 7.5, 1.2 Hz, 1H), 7.47-7.35 (m, 3H), 3.75 (s, 6H); ^{13}C{^{1}H} NMR (100 MHz, $CDCl_3$): δ 166.3, 133.6, 132.1, 130.2, 128.6, 122.4, 117.4, 52.5; HRMS (ESI)

m/z: $[M + Na]^+$ Calcd for $C_{11}H_{10}NaBrIO_4$ 434.8700; Found 434.8685;$[M + H]^+$ Calcd for $C_{11}H_{11}BrIO_4$ 412.8880; Found 412.8876.

Note: Compound **11** was previously reported as an intermediate product, without any further purification and characterization.[10]

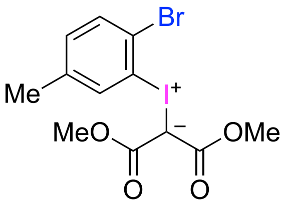

12

Product **12** was prepared by following the general procedure (A) from reaction of compound **2** (457 mg, 1.1 mmol, 1.1 equiv.) and dimethyl malonate (116 μL, 1.0 mmol, 1.0 equiv.) together with KF (337 mg, 6.0 mmol, 6.0 equiv.) in 5 mL CH_3CN. The product was obtained as a white solid (277 mg, 59%): mp 126-128 °C; ^{1}H NMR (400 MHz, $CDCl_3$): δ 7.45 (d, *J* = 8.0 Hz, 1H), 7.18 (d, *J* = 7.9 Hz, 1H), 7.10 (s, 1H), 3.76 (s, 6H), 2.36(s, 3H); $^{13}C\{^1H\}$ NMR (100 MHz, $CDCl_3$): δ 166.4, 141.3, 133.2, 133.1, 128.5, 118.8, 117.0, 52.5, 21.3; HRMS (ESI) *m/z*: $[M + Na]^+$ Calcd for $C_{12}H_{12}NaBrIO_4$ 448.8856; Found 448.8885;$[M + H]^+$ Calcd for $C_{12}H_{13}BrIO_4$ 426.9037; Found 426.9072.

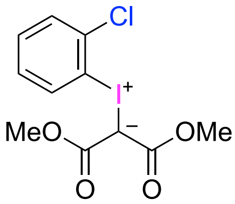

13

Product **13** was prepared by following the general procedure (A) from reaction of compound **4** (392 mg, 1.1 mmol, 1.1 equiv.) and dimethyl malonate (116 µL, 1.0 mmol, 1.0 equiv.) together with KF (337 mg, 6.0 mmol, 6.0 equiv.) in 5 mL CH_3CN. The product was obtained as a white solid (264 mg, 65%): mp 125-127 °C; ^1H NMR (400 MHz, $CDCl_3$): δ 7.50-7.37 (m, 4H), 3.75 (s, 6H); HRMS (ESI) *m/z*: $[M +Na]^+$ Calcd for $C_{11}H_{10}ClINaO_4$ 390.9205; Found 390.9197; $[M +H]^+$ Calcd for $C_{11}H_{11}ClIO_4$ 368.9386; Found 368.9383.

Note: Compound **13** was previously reported, mp 140-141 °C.[8] Our procedure resulted in a better yield of the product.

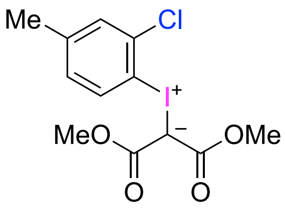

14

Product **14** was prepared by following the general procedure (A) from reaction of compound **5** (408 mg, 1.1 mmol, 1.1 equiv.) and dimethyl malonate (116 µL, 1.0 mmol, 1.0 equiv.) together with KF (337 mg, 6.0 mmol, 6.0 equiv.) in 5 mL CH_3CN. The product was obtained as a white solid (232 mg, 55%): mp 122-124 °C; ^1H NMR (400 MHz, $CDCl_3$): δ 7.32-7.30 (m, 2H), 7.20 (d, J = 8.2 Hz, 1H), 3.76 (s, 6H), 2.41(s, 3H); $^{13}C\{^1H\}$ NMR (100 MHz, $CDCl_3$): δ 166.3, 143.1, 132.7, 131.0, 130.6, 128.5, 110.4, 52.4, 20.9; HRMS (ESI) *m/z*: $[M +Na]^+$ Calcd for $C_{12}H_{12}ClINaO_4$ 404.9362; Found 404.9399; $[M +H]^+$ Calcd for $C_{12}H_{13}ClIO_4$ 382.9542; Found 382.9572.

*General procedure (B) for ylides **15** and **16**:*[7]

To a 25 mL flask containing KOH (337 mg, 6.0 mmol, 6.0 equiv.) and the malonate ester (1.2 mmol, 1.2 equiv.) under argon atmosphere, 7 mL dry CH_2Cl_2 was added. The heterogeneous mixture was placed in a water bath at 25 °C. Then it was stirred vigorously for 5 minutes to form a milky white suspension, followed by the addition of the (diacetoxyiodo)arene (1.0 mmol, 1.0 equiv.) in one portion. The reaction mixture was

stirred at room temperature, and a yellow heterogeneous mixture gradually formed. After 2 hours, it was filtered and then concentrated under reduced pressure at room temperature. A yellow mixture of solid and oil was obtained, which was stirred in 20 mL of hexane, to form a pale yellow solid. It was filtered and then dried under vacuum.

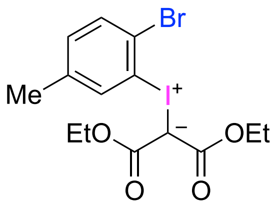

15

Product **15** was prepared by following the general procedure (B) from reaction of compound **2** (415 mg, 1.0 mmol, 1.0 equiv.) and diethyl malonate (183 μL, 1.2 mmol, 1.2 equiv.) together with KF (337 mg, 6.0 mmol, 6.0 equiv.) in 7 mL CH_2Cl_2. The product was obtained as a pale yellow solid (127 mg, 28%): mp 94-96 °C; ^{1}H NMR (400 MHz, $CDCl_3$): δ 7.37 (d, J = 7.7 Hz, 1H), 7.10 (d, J = 7.2 Hz, 1H), 7.05 (s, 1H), 4.13 (d, J = 6.6 Hz, 4H), 2.29 (s, 3H), 1.22-1.20 (m, 6H); $^{13}C\{^1H\}$ NMR (100 MHz, $CDCl_3$): δ 166.0, 141.1, 133.1, 133.1, 128.8, 118.8, 117.1, 61.0, 21.2, 14.7; HRMS (ESI) *m/z*: $[M + H]^+$ Calcd for $C_{14}H_{17}BrIO_4$ 454.9350; Found 454.9389.

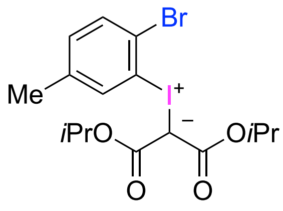

16

Product **16** was prepared by following the general procedure (B) from reaction of compound **2** (415 mg, 1.0 mmol, 1.0 equiv.) and diisopropyl malonate (228 μL, 1.2 mmol, 1.2 equiv.) together with KF (337 mg,

6.0 mmol, 6.0 equiv.) in 7 mL CH_2Cl_2. The product was obtained as a pale yellow solid (313 mg, 65%): mp 106-108 ºC; ^1H NMR (400 MHz, $CDCl_3$): δ 7.46 (d, J = 8.0 Hz, 1H), 7.18 (d, J = 8.0 Hz, 1H), 7.14 (s, 1H), 5.06 (septet, J = 6.3 Hz, 2H), 2.37(s, 3H); 1.26 (d, J = 6.2 Hz, 12H); $^{13}C\{^1H\}$ NMR (100 MHz, $CDCl_3$): δ 165.4, 140.9, 133.1, 133.0, 129. 0, 118.9, 117.4. 68.0, 22.3, 21.2; HRMS (ESI) *m/z*: $[M +H]^+$ Calcd for $C_{16}H_{21}BrIO_4$ 482.9663; Found 482.9697.

6. Stability of iodonium ylides

Iodonium ylides **11-16** slowly decompose in solution at room temperature and at low temperature; however, clean NMR and mass spectra can be obtained by taking spectra immediately after dissolving solid compounds in $CDCl_3$ or CD_3CN. We have tested the stability of ylides **11**, **12**, and **13** in comparison with the unsubstituted reference ylide, $PhIC(CO_2Me)_2$, in the solid state at room temperature. It is known from the literature that iodonium ylides, $ArIC(CO_2Me)_2$, cleanly decompose with formation of 2ArI and the product of carbene dimerization, $(MeOCO)_2C=C(COOMe)_2$.[7-9] The degree of decomposition can be conveniently measured by integration of the MeO signals corresponding to the original ylide (at 3.76-3.76 ppm) and $(MeOCO)_2C=C(COOMe)_2$ (3.87-3.88 ppm) in the partially decomposed sample. Samples of solid ylides were placed on the lab bench for 4 weeks. The NMR has shown good stability for ylide **11** (Figure 2S) but some degradation was observed for ylide **13** (Figure 3S, 2.8:5.0 MeO ratio of decomposed to original ylide; 36% decomposition), and significant degradation and color change was observed for ylide **12** (Figure 4S, 5.0:1.2 MeO ratio of decomposed to original ylide; 81% decomposition). In comparison, significant decomposition was observed after 4 weeks for the unsubstituted reference ylide, $PhIC(CO_2Me)_2$ (Figure 5S, 9.66:3.76 MeO ratio of decomposed to original ylide; 72% decomposition). Ylides **11-16** can be stored for several months in a refrigerator without noticeable degradation.

*NMR spectra of reference ylide and ylides **11**, **12** and **13** in $CDCl_3$ in comparison with the unsubstituted reference ylide, $PhIC(CO_2Me)_2$.*

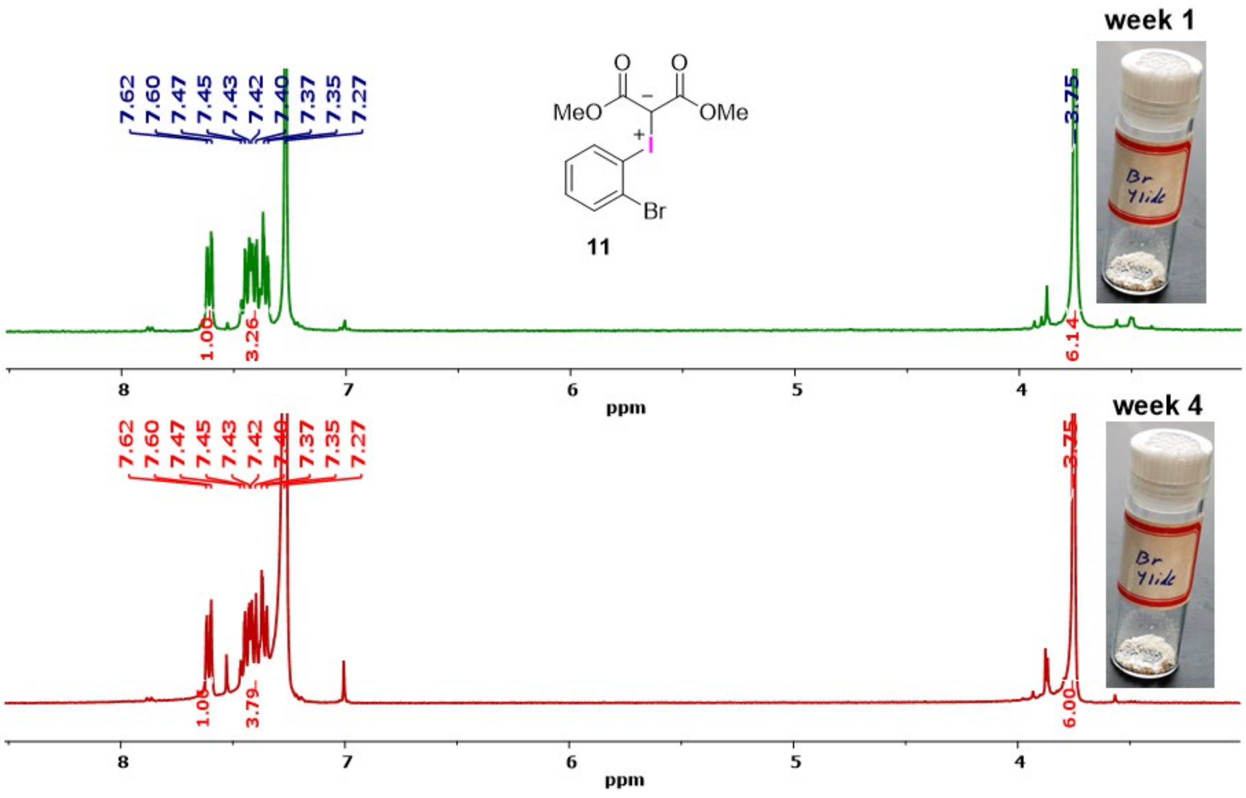

Figure 2S: ^{1}H NMR spectra of ylide **11** immediately after preparation and after 4 weeks of storage at room temperature.

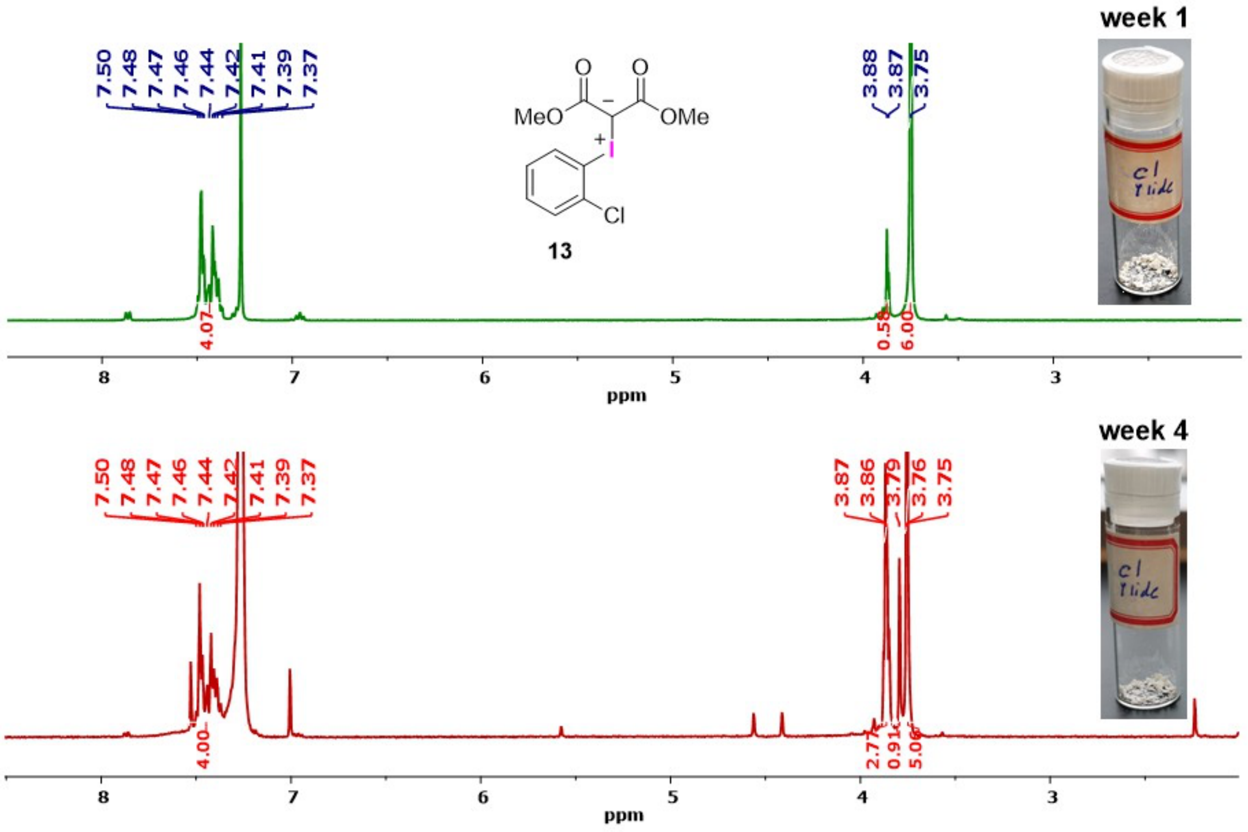

Figure 3S: ^{1}H NMR spectra of ylide **13** immediately after preparation and after 4 weeks of storage at room temperature.

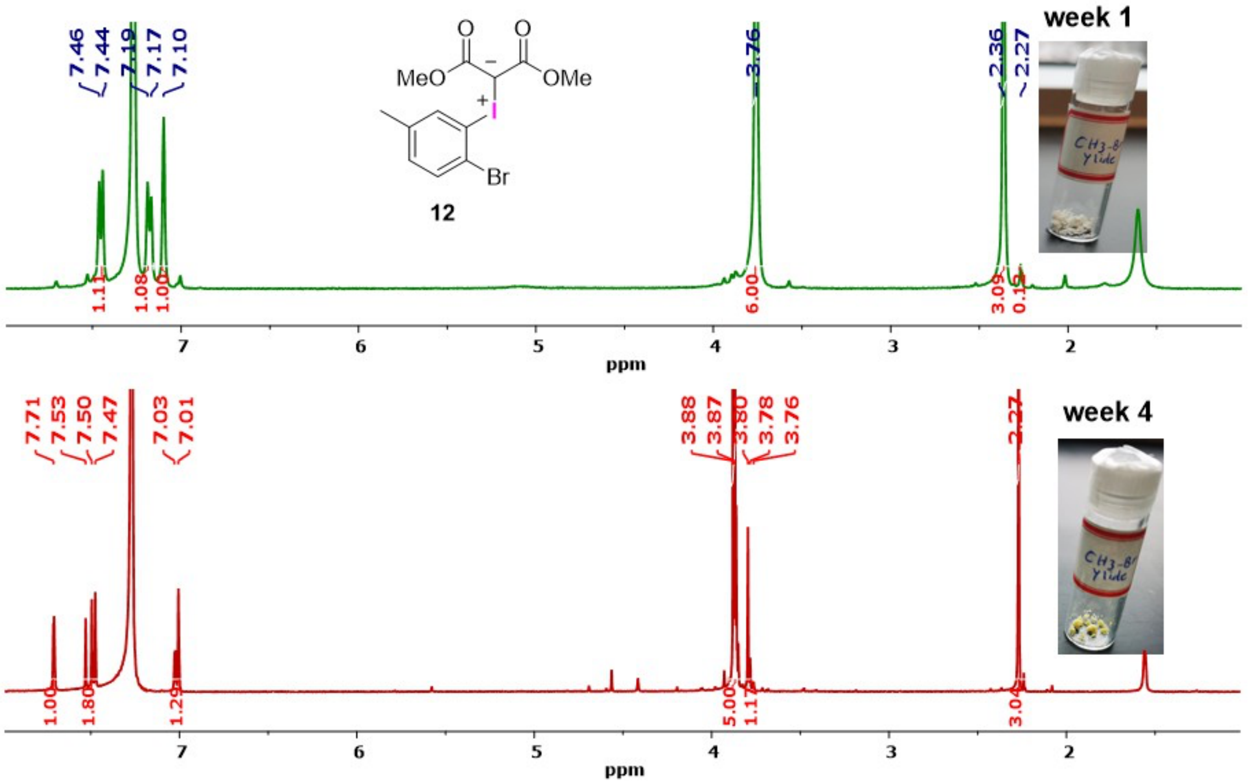

Figure 4S: ^{1}H NMR spectra of ylide **12** immediately after preparation and after 4 weeks of storage at room temperature.

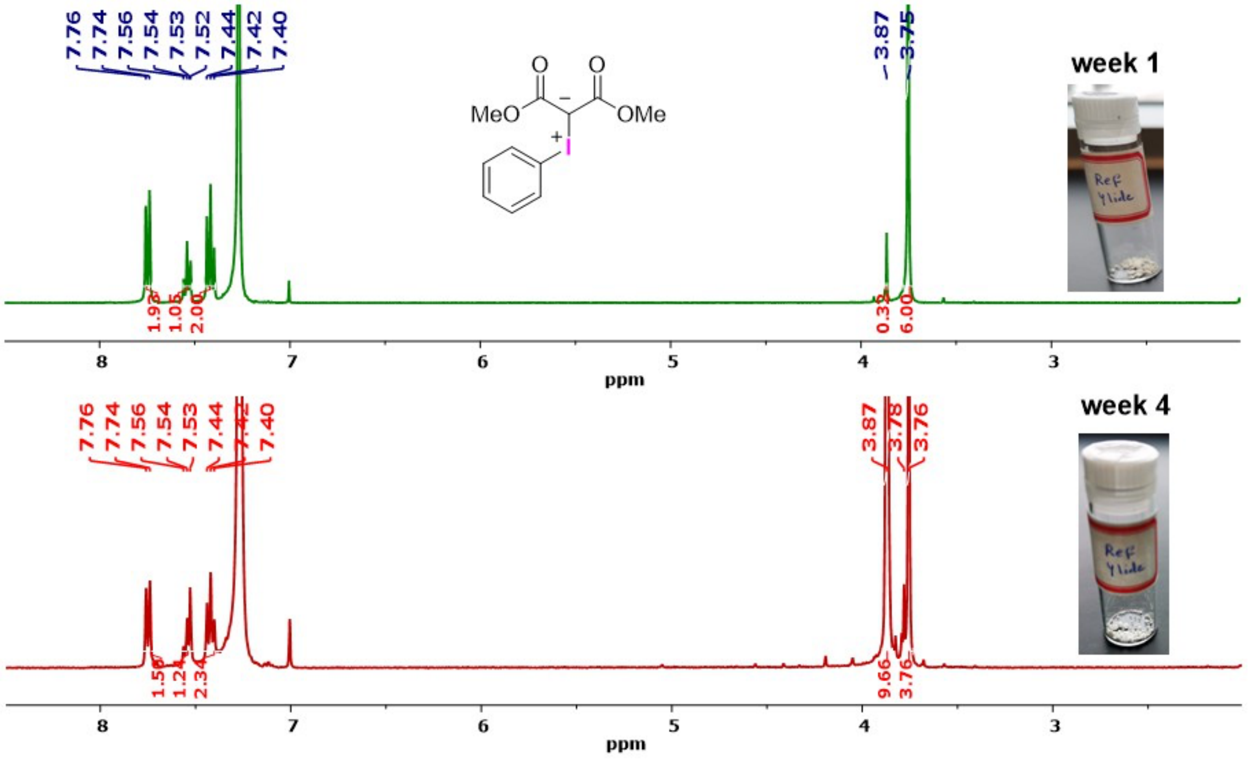

Figure 5S: ^{1}H NMR spectra of the unsubstituted reference ylide, $PhIC(CO_2Me)_2$, immediately after preparation and after 4 weeks of storage at room temperature. NOTE: the second product of decomposition, PhI, completely evaporated after storage for 4 weeks at room temperature.

7. Cyclopropanation of styrenes with iodonium ylide 11

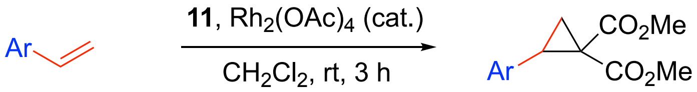

17-20

General procedure:[7,9]

1,1-Cyclopropanes diesters **17-20** were prepared according to the following general procedure. To a 25 ml flask containing appropriate styrene (5.0 mmol, 5.0 equiv.) and the catalyst, Rh(II)acetate dimer (2.2 mg, 5.0 µmol, 0.5 mol %) under argon, 7 ml dry CH_2Cl_2 added. Iodonium ylide **11** (1.0 mmol, 1.0 equiv.) was

added in 3 portions during 10 minutes at room temperature. Reaction mixture was stirred for 3 h. The solution was then concentrated and purified by column chromatography on silica gel (10% EtOAc/hexane).

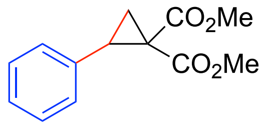

17

Product **17** was prepared by following the general procedure from reaction of iodonium ylide **11** (413 mg, 1.0 mmol, 1.0 equiv.) and styrene (575 μL, 5.0 mmol, 5.0 equiv.), together with Rh(II)acetate dimer (2.2 mg, 5.0 μmol, 0.5 mol %) in 7 mL CH_2Cl_2. After purification by using column chromatography on silica gel (10% EtOAc/hexane) the product was obtained as a colorless oil (164 mg, 70%): ^1H NMR (400 MHz, $CDCl_3$): δ 7.22-7.11 (m, 5H), 3.72 (s, 3H), 3.29 (s, 3H), 3.16 (t, J = 8.7 Hz, 1H), 2.13 (dd, J = 7.9, 5.2 Hz, 1H), 1.68 (dd, J = 9.2, 5.2 Hz, 1H); HRMS (ESI) m/z: $[M+Na]^+$ Calcd for $C_{13}H_{14}NaO_4$ 257.0785; Found 257.0789.

Note: The NMR data of product **17** was consistent with the previously reported data.[7, 9,11-13] The HRMS of product **17** was not reported in previous works.

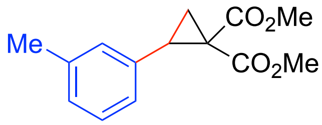

18

Product **18** was prepared by following the general procedure from reaction of iodonium ylide **11** (413 mg, 1.0 mmol, 1.0 equiv.) and 3-methylstyrene (664 μL, 5.0 mmol, 5.0 equiv.), together with Rh(II)acetate dimer (2.2 mg, 5.0 μmol, 0.5 mol %) in 7 mL CH_2Cl_2. After purification by using column chromatography on silica gel (10% EtOAc/hexane) the product was obtained as a colorless oil (179 mg, 72%): ^1H NMR (400 MHz, $CDCl_3$): δ 7.15 (t, J = 7.5 Hz, 1H), 7.04 (s, 1H), 7.02(s, 1H), 6.97 (d, J = 7.6 Hz, 1H), 3.79 (s, 3H), 3.38 (s, 3H), 3.19 (t, J = 8.7 Hz, 1H), 2.31 (s, 3H), 2.18 (dd, J = 8.0, 5.2 Hz, 1H), 1.73 (dd, J = 9.2, 5.1 Hz, 1H); HRMS (ESI) m/z: $[M+Na]^+$ Calcd for $C_{14}H_{16}NaO_4$ 271.0941; Found 271.0937.

Note: The analytical data of product **18** was consistent with the previously reported data.[9,11-13]

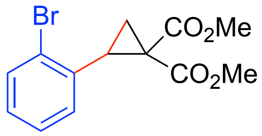

19

Product **19** was prepared by following the general procedure from reaction of iodonium ylide **11** (413 mg, 1.0 mmol, 1.0 equiv.) and 2-bromostyrene (627 µL, 5.0 mmol, 5.0 equiv.), together with Rh(II)acetate dimer (2.2 mg, 5.0 µmol, 0.5 mol %) in 7 mL CH_2Cl_2. After purification by using column chromatography on silica gel (10% EtOAc/hexane) the product was obtained as a colorless oil (184, mg, 59%): ^{1}H NMR (400 MHz, $CDCl_3$): : δ 7.55 (dd, *J* = 7.8, 1.1 Hz, 1H), 7.23 (td, *J* = 7.5, 1.1, 1H), 7.14-7.08 (m, 2H), 3.82 (s, 3H), 3.36 (s, 3H), 3.34 (t, *J* = 8.7 Hz, 1H), 2.26 (dd, *J* = 8.2, 5.2 Hz, 1H), 1.80 (dd, *J* = 9.2, 5.2 Hz, 1H); HRMS (ESI) *m/z*: $[M +Na]^+$ Calcd for $C_{13}H_{13}BrNaO4$ 334.9890; Found 334.9920.

Note: The NMR data of product **19** was consistent with the previously reported data.[7,13] The HRMS of product **19** was not reported in previous works.

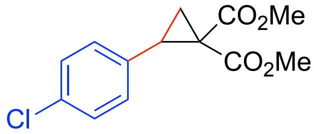

20

Product **20** was prepared by following the general procedure from reaction of iodonium ylide **11** (413 mg, 1.0 mmol, 1.0 equiv.) and 4-chlorostyrene (600 µL, 5.0 mmol, 5.0 equiv.), together with Rh(II)acetate dimer (2.2 mg, 5.0 µmol, 0.5 mol %) in 7 mL CH_2Cl_2. After purification by using column chromatography on silica gel (10% EtOAc/hexane) the product was obtained as a yellow oil (202 mg, 75%): ^{1}H NMR (400 MHz, $CDCl_3$): δ 7.17 (d, *J* = 8.6 Hz, 2H), 7.06 (d, *J* = 8.5 Hz, 2H), 3.72 (s, 3H), 3.34 (s, 3H), 3.11(t, *J* = 8.7, 1H), 2.08(dd, *J* = 8.0, 5.3 Hz, 1H), 1.67 (dd, *J* = 9.2, 5.3 Hz, 1H); HRMS (ESI) *m/z*: $[M +Na]^+$ Calcd for $C_{13}H_{13}ClNaO_4$ 291.0395; Found 291.0401.

Note: The NMR data of product **20** was consistent with the previously reported data. [9,11-13] The HRMS of product **20** was not reported in previous works.

References

(1) Fujie, M.; Mizufune, K.; Nishimoto, Y.; Yasuda, M. 1-Fluoro-1-sulfonyloxylation of Alkenes by Sterically and Electronically Tuned Hypervalent Iodine: Regression Analysis toward 1,1-Heterodifunctionalization. *Org. Lett.* **2023**, *25*, 766-770.

(2) (a) Chun, J. -H.; Lu, -S.; Lee, Y. S.; Pike, V. W. Fast and High-Yield Microreactor Syntheses of ortho-Substituted[^{18}F] Fluoroarenes from Reactions of [^{18}F] Fluoride Ion with Diaryliodonium Salts. *J. Org. Chem.***2010**, *10*, 3332-3338. (b) Haskali, M. B.; Telu, S.; Lee, Y. S.; Morse, C. L.; Lu, S.; Pike, V.W. An investigation of (diacetoxyiodo)arenes as precursors for preparing no-carrier-added [^{18}F]fluoroarenes from cyclotronproduced [^{18}F]fluoride ion. *J. Org. Chem*. **2016**, *81*, 297−302.

(3) Izquierdo, S.; Bouvet, S.; Wu, Y.; Molina, S.; Shafir, A. The Coming of Age in Iodane-Guided ortho-CH Propargylation: From Insight to Synthetic Potential. *Chem. Eur. J.* **2018**, *24*, 15517- 15521.

(4) Chun, J.-H.; Pike. V. W. Regiospecific Syntheses of Functionalized Diaryliodonium Tosylates via [Hydroxy(tosyloxy)iodo]arenes Generated in Situ from (Diacetoxyiodo)arenes *J. Org. Chem*. **2012**, *77*, 1931-1938.

(5) Koser, G.F.; Wettach, R. H. Hypervalent organoiodine. Reactions of silver arylsulfonates with Iodosobenzene dichloride. *J. Org. Chem*. **1977**, *42*, 1476-1478.

(6) Chen, W. W.; Artigues, M.; Font-Bardia, M.; Cuenca, A. B.; Shafir, A. Cyclic homo- and hetero-halogen di-λ3-diarylhalonium structures. *J. Am. Chem. Soc.* **2023**, *145*, 13796-13804.

(7) Goudreau, S. R.; Marcoux, D.; Charette, A. B. General Method for the Synthesis of Phenyliodonium Ylides from Malonate Esters: Easy Access to 1,1-Cyclopropane Diesters. *J. Org. Chem.* **2009**, *74*, 470-473.

(8) Xu, W.; Li, P.; Li, Y.; Kou. Z.; Li, K.; Li, H.; Ali, S.; Pan, J.; Wang, Z.; Zheng, H. Iodonium(III) Ylide: An Iodoalkylation Reagent with Aryne. *J. Org. Chem.* **2025**, *90*, 2372−2385.

(9) Zhu, C.; Yoshimura, A.; Ji, L.; Wei, Y.; Nemykin, V. N.; Zhdankin, V.V. Design, Preparation, X-ray Crystal Structure, and Reactivity of o-Alkoxyphenyliodonium Bis(methoxycarbonyl)methanide, a Highly Soluble Carbene Precursor. *Org. Lett*., **2012**, *14*, 3170-3173.

(10) Hartmann, M.; Li, Y.; Mück-Lichtenfeld, C.; Studer, A. Generation of Aryl Radicals through Reduction of Hypervalent Iodine(III) Compounds with TEMPONa: Radical Alkene Oxyarylation. *Chem. Eur. J.* **2016**, *22*, 3485-3490.

(11) Deng, C.; Wang, L. J.; Zhu, J.; Tang. Y. A Chiral Cage-like Copper(I) Catalyst for Highly Enantioselective Synthesis of 1,1-Cyclopropane Diesters. *Angew. Chem. Int. Ed*. **2012**, *51*, 11620-11623.

(12) Nishimura, T.; Maeda, Y.; Hayashi, T. Asymmetric Cyclopropanation of Alkenes with Dimethyl Diazomalonate Catalyzed by Chiral Diene–Rhodium Complexes. *Angew. Chem. Int. Ed*. **2010**, *49*, 7324 – 7327.

(13) Huang, H.; Gao, M.; Yang, Z.; Han,W.; Wei, Z.; Li, Z.; Xu, B. 1,3-Difunctionalization of Donor-Acceptor Cyclopropanes Enabled by Copper Nitrate: A Direct Approach to α-Halonitrates *Org. Lett.* **2024**, *26*, 9659-9664.

NZ-BrAOc-1 1 1 D:\nmrdata\vzgroup

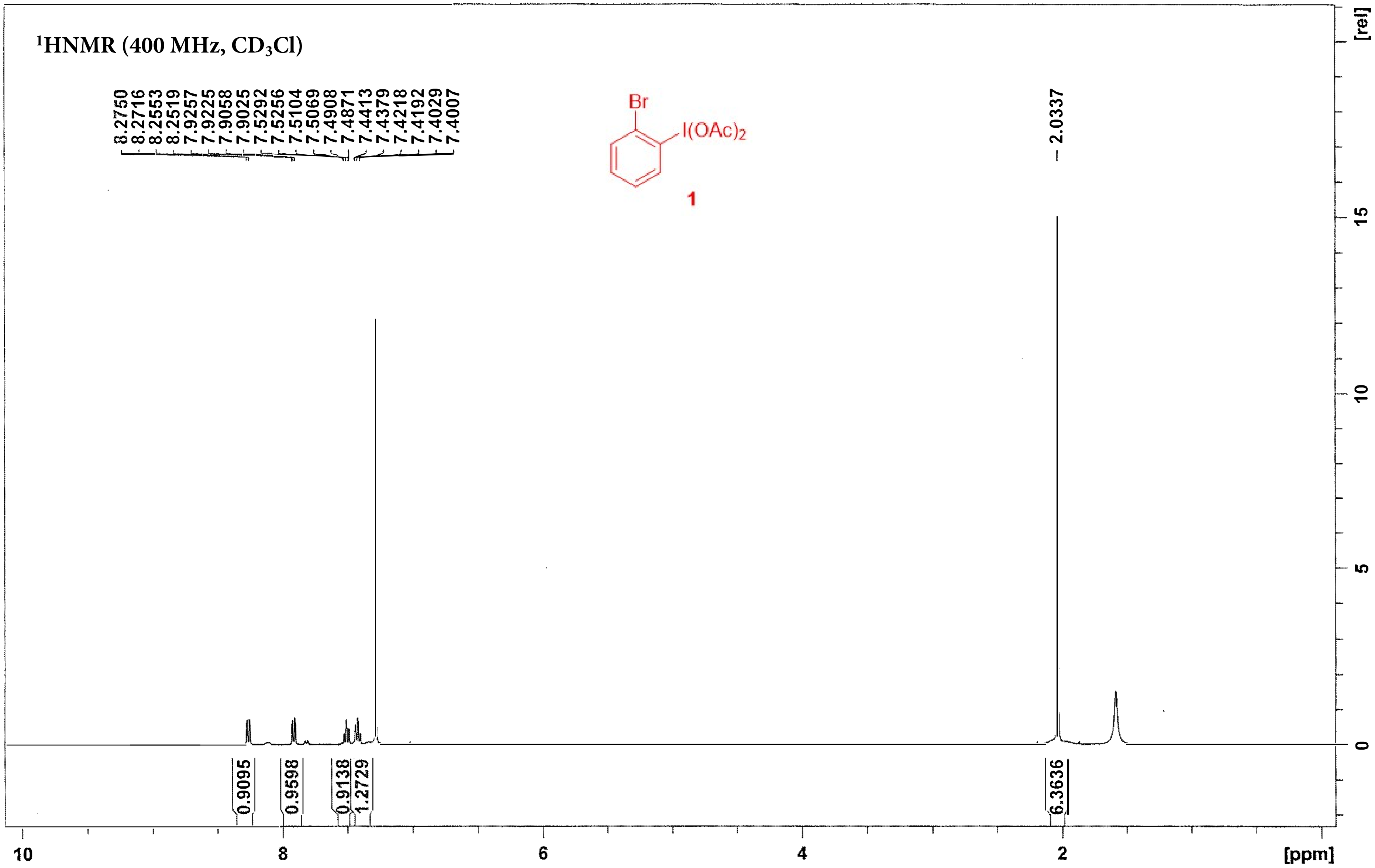

NZ-Br-CH3-OAc 1 1 D:\nmrdata\vzgroup

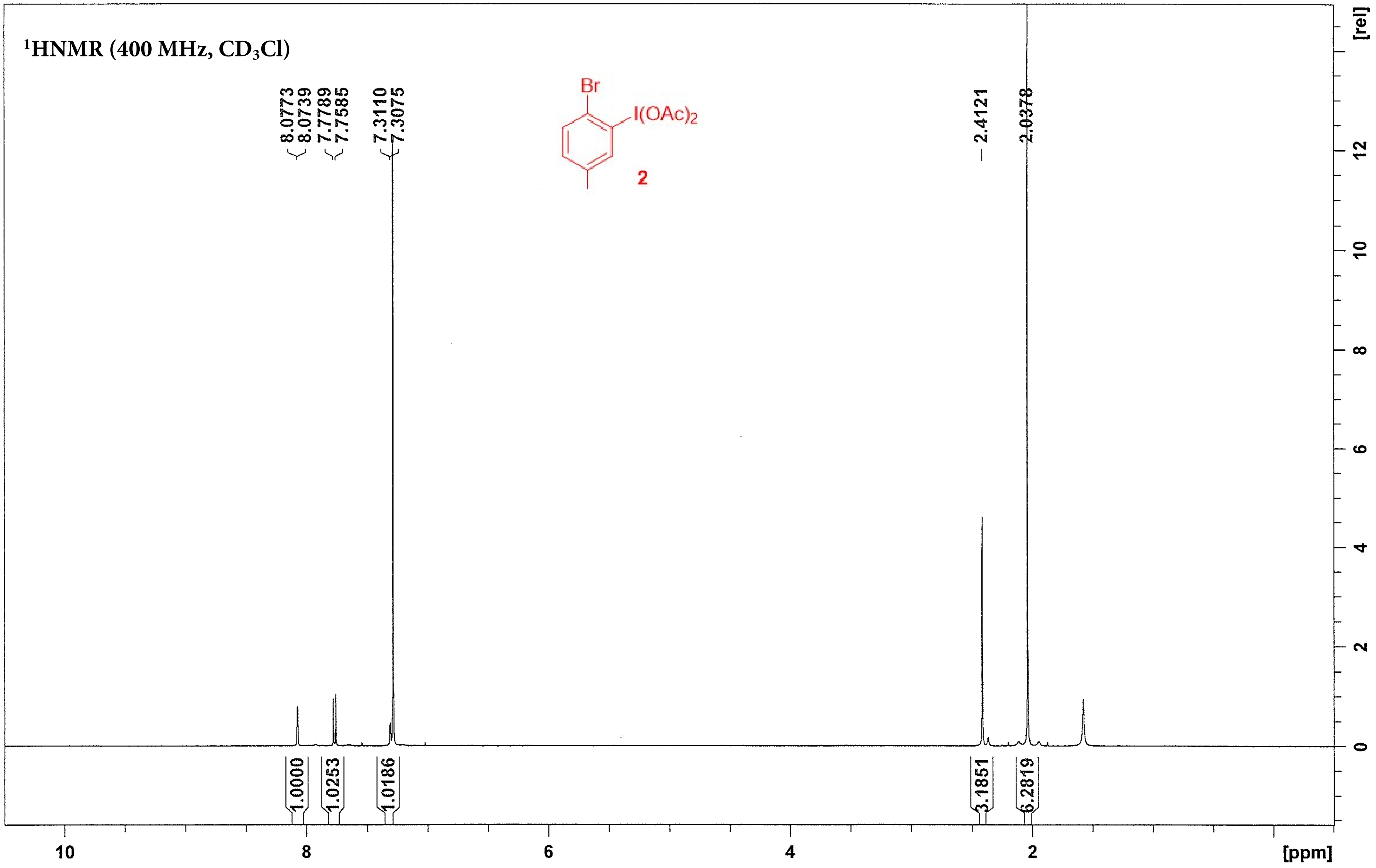

BH-BrCH2-I-OAc 1 1 D:\nmrdata\vzgroup

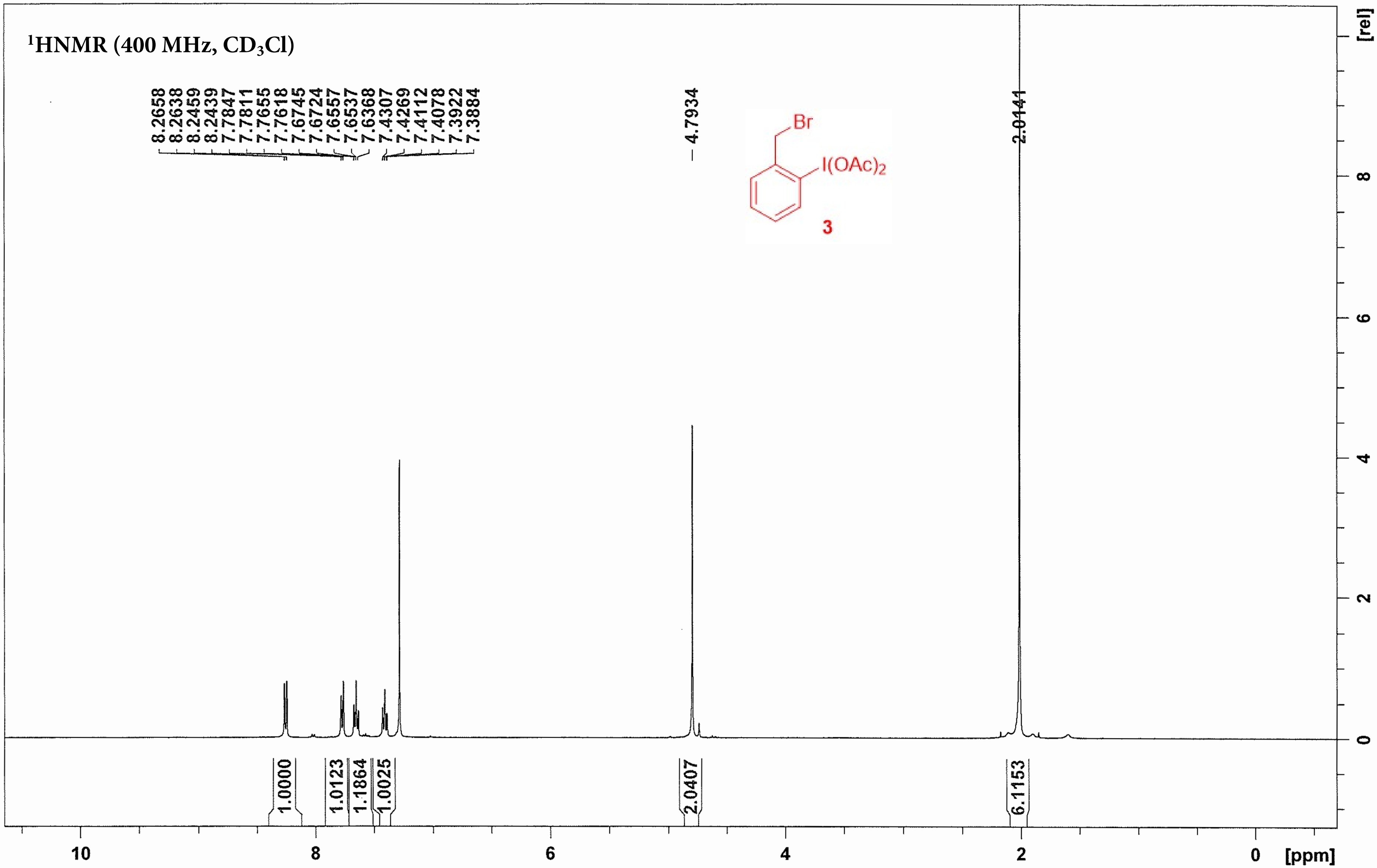

NZ-I-Cl-OAC-4h 1 1 C:\Data\vzgroup

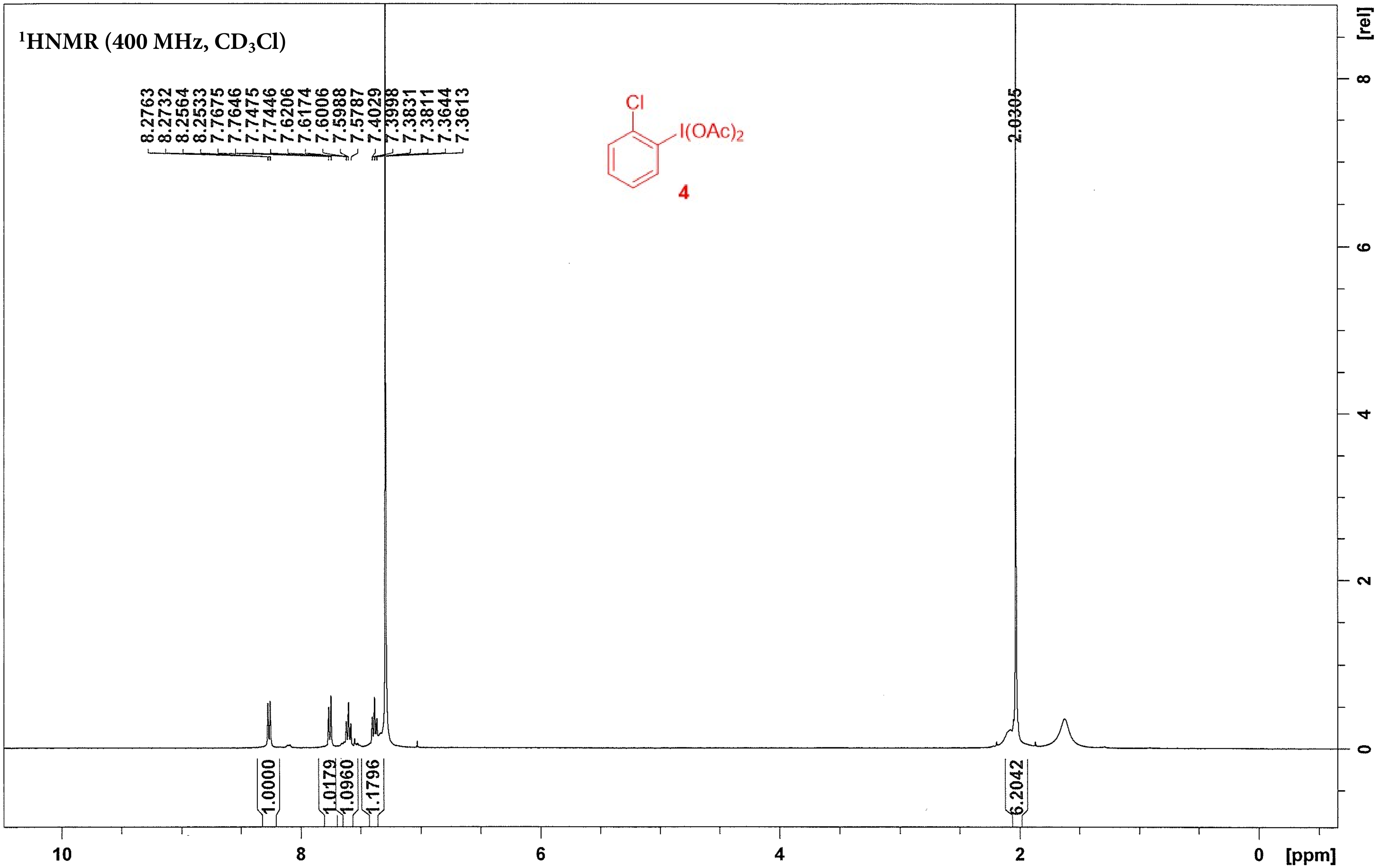

BH-ClCH3-OAc-I 1 1 D:\nmrdata\vzgroup

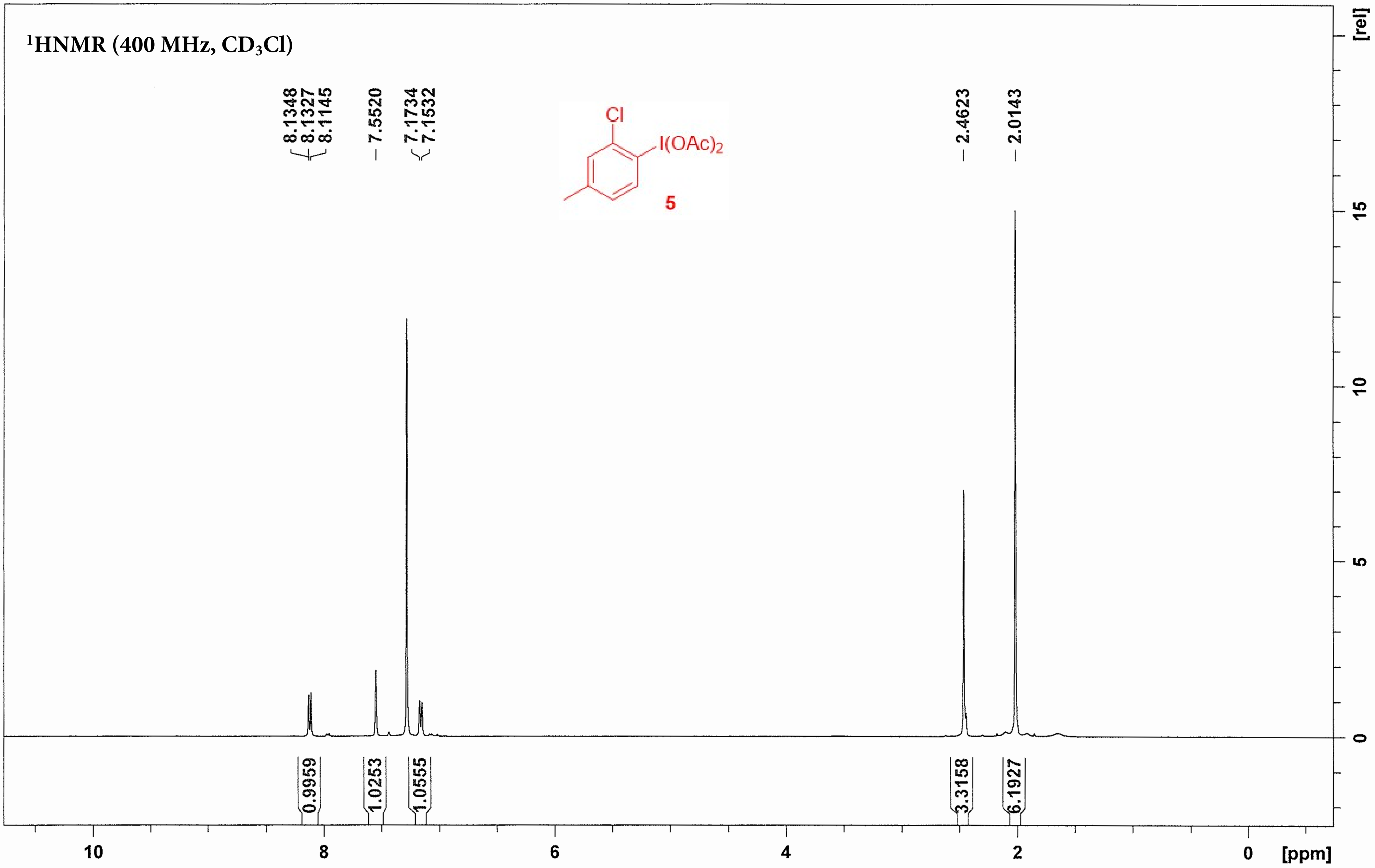

BH-ClCH3-IOAc-C 1 1 D:\nmrdata\vzgroup

$^{13}C\{^{1}H\}$ NMR (100 MHz, $CDCl_3$)

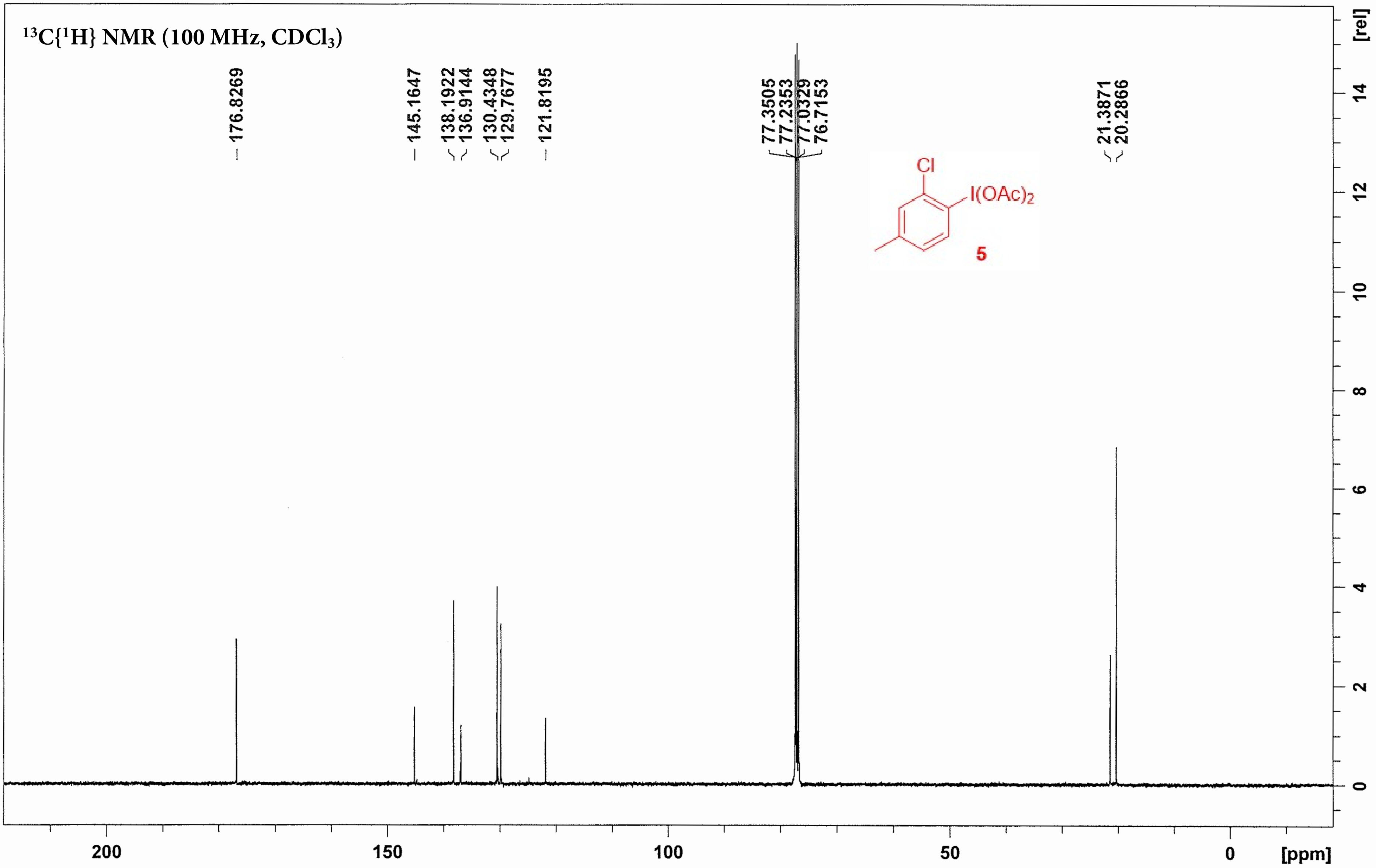

NZ-I-Br-OTs 1 1 C:\Data\vzgroup

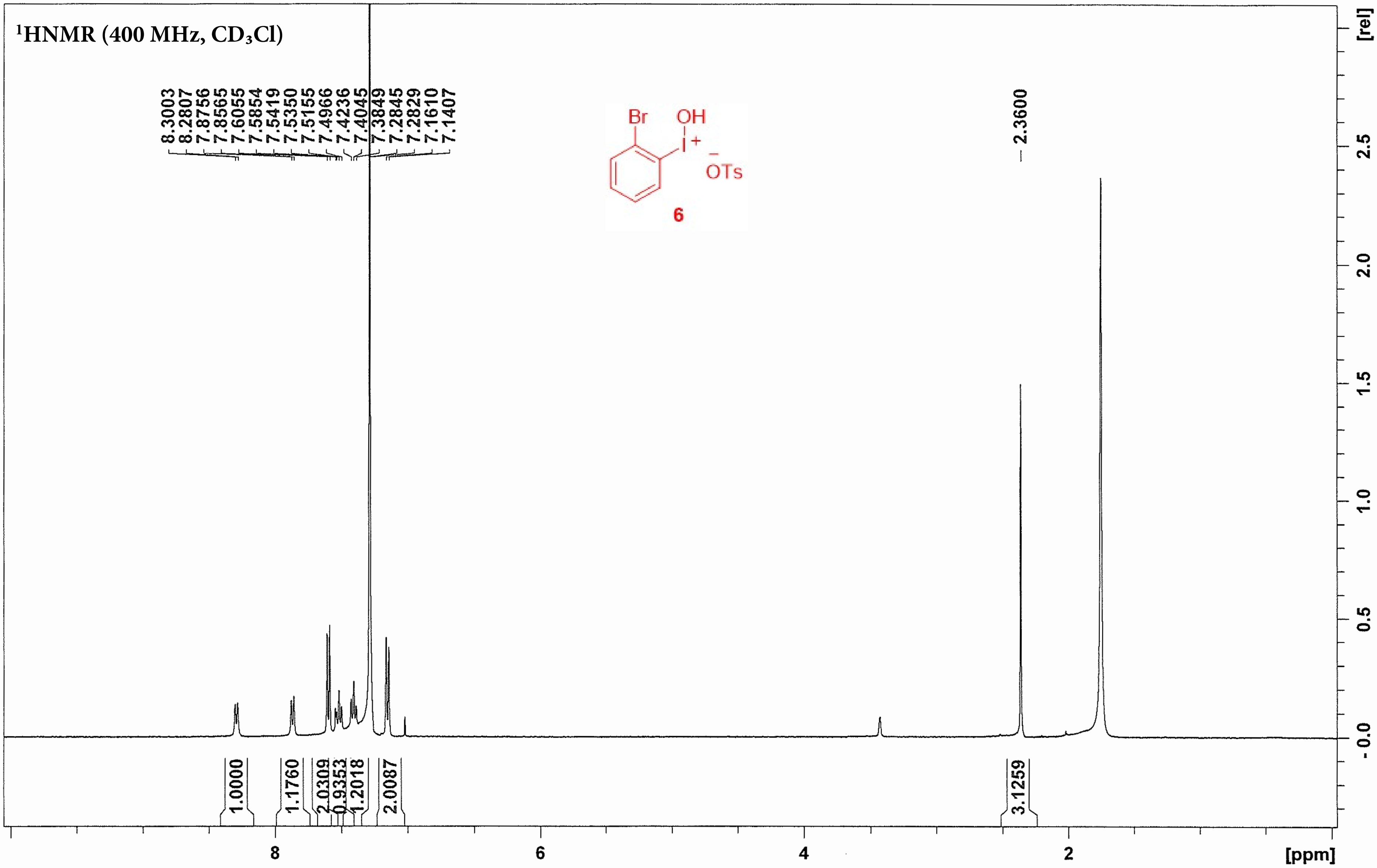

CH3

BH-Br-OTs 1 1 D:\nmrdata\vzgroup

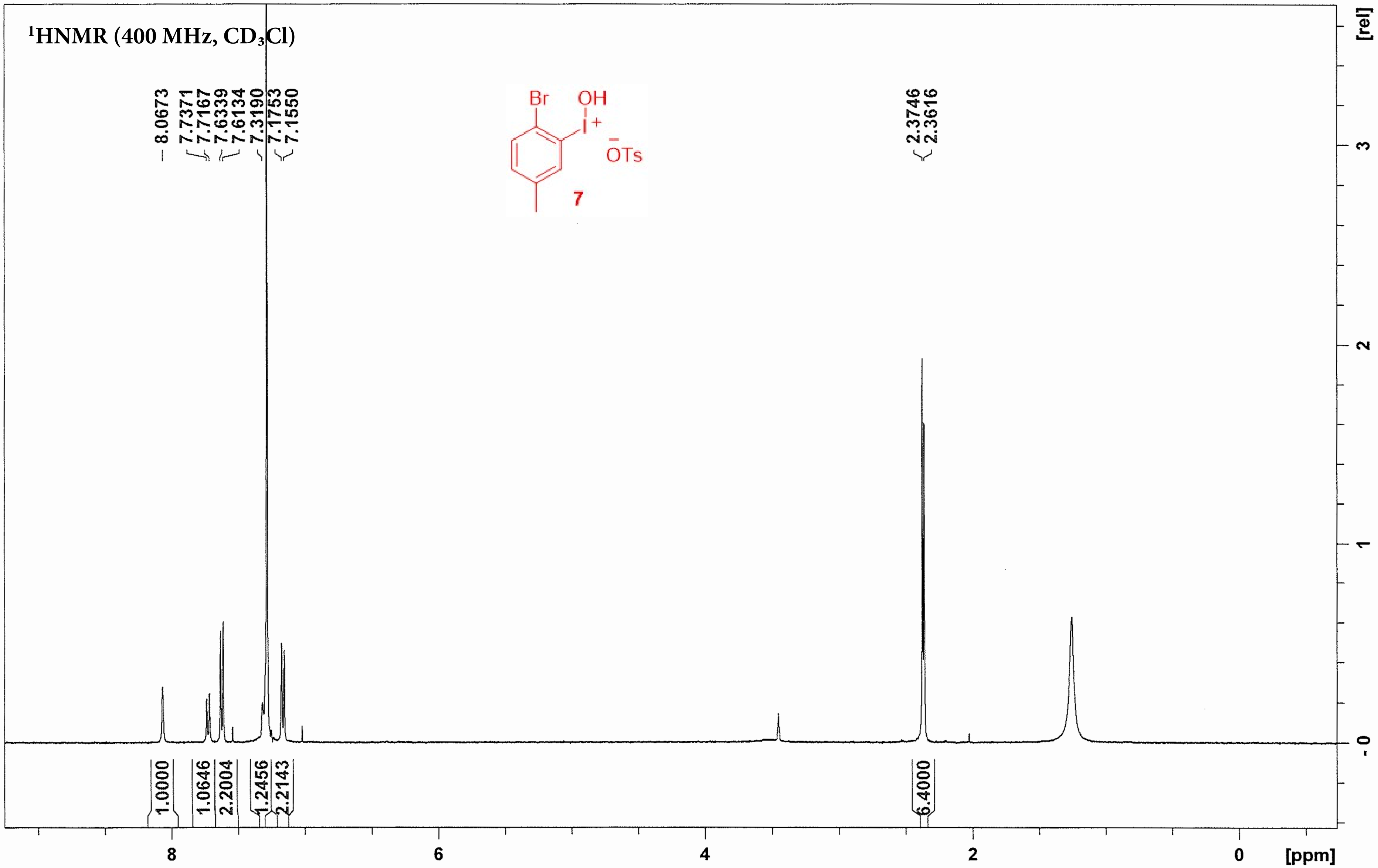

BH-CH3BrIPh-OTs-C 1 1 D:\nmrdata\vzgroup

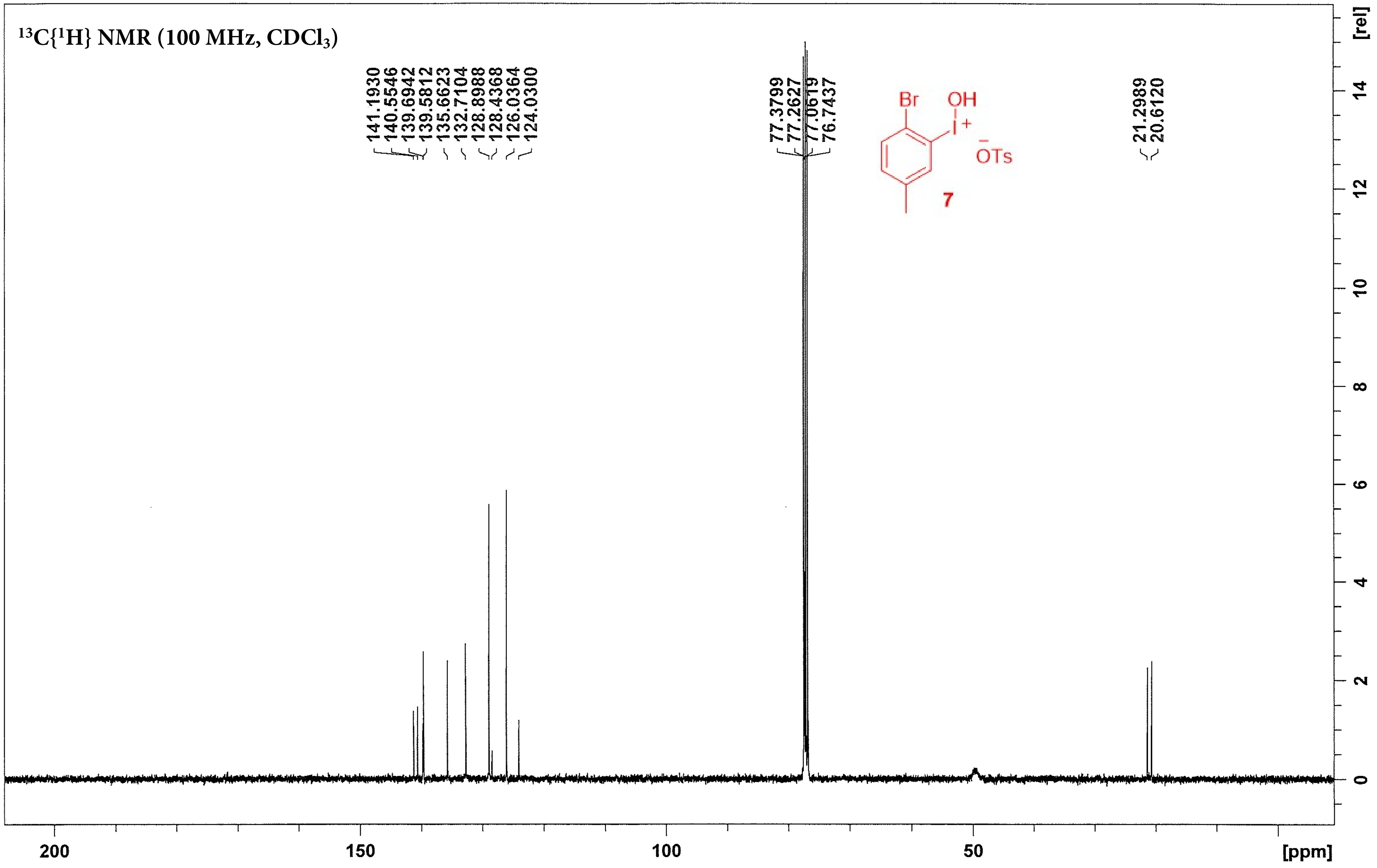

BH-CH2Br-IOTs-2 1 1 D:\nmrdata\vzgroup

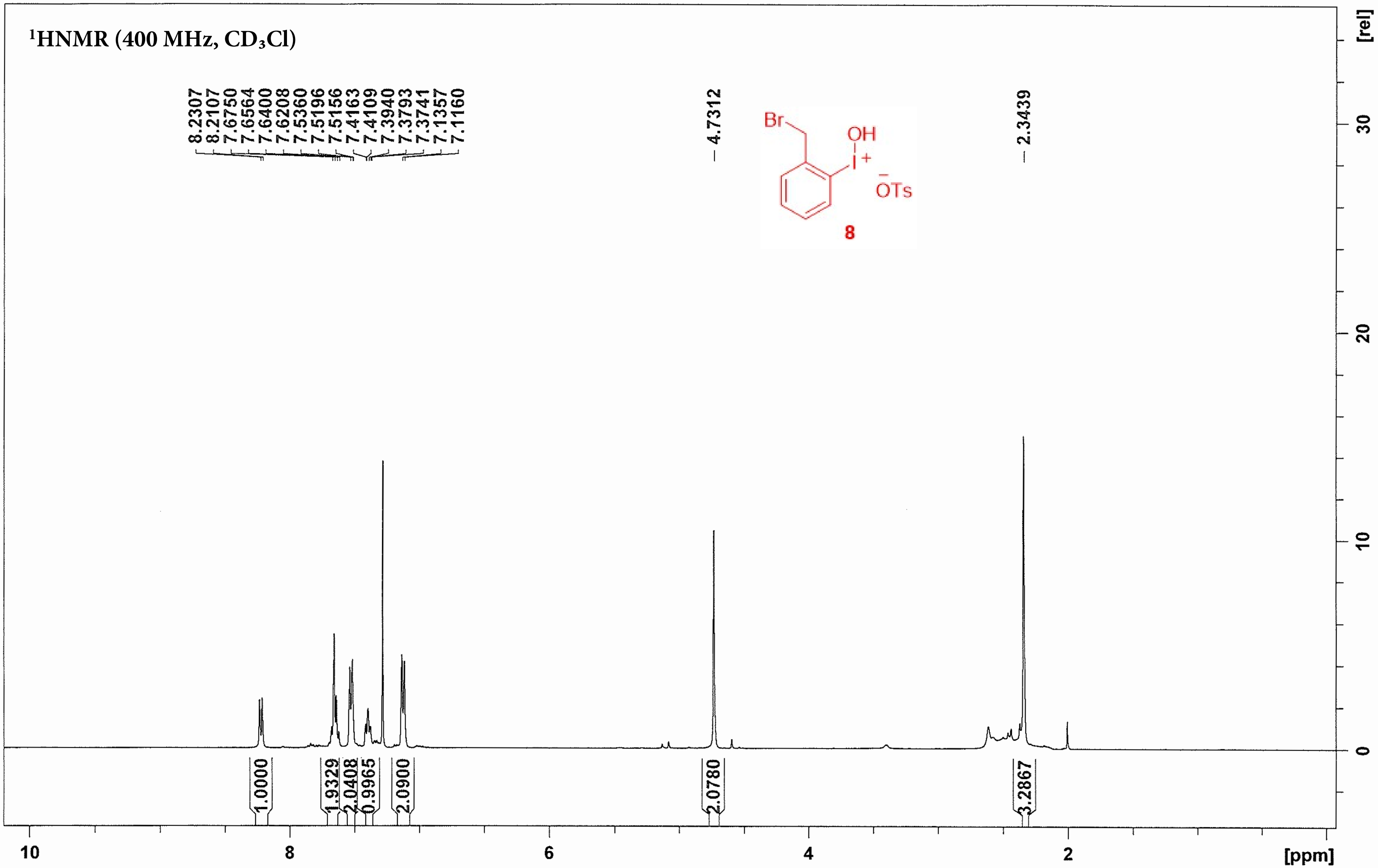

BH-CH2Br-OTs-C 1 1 D:\nmrdata\vzgroup

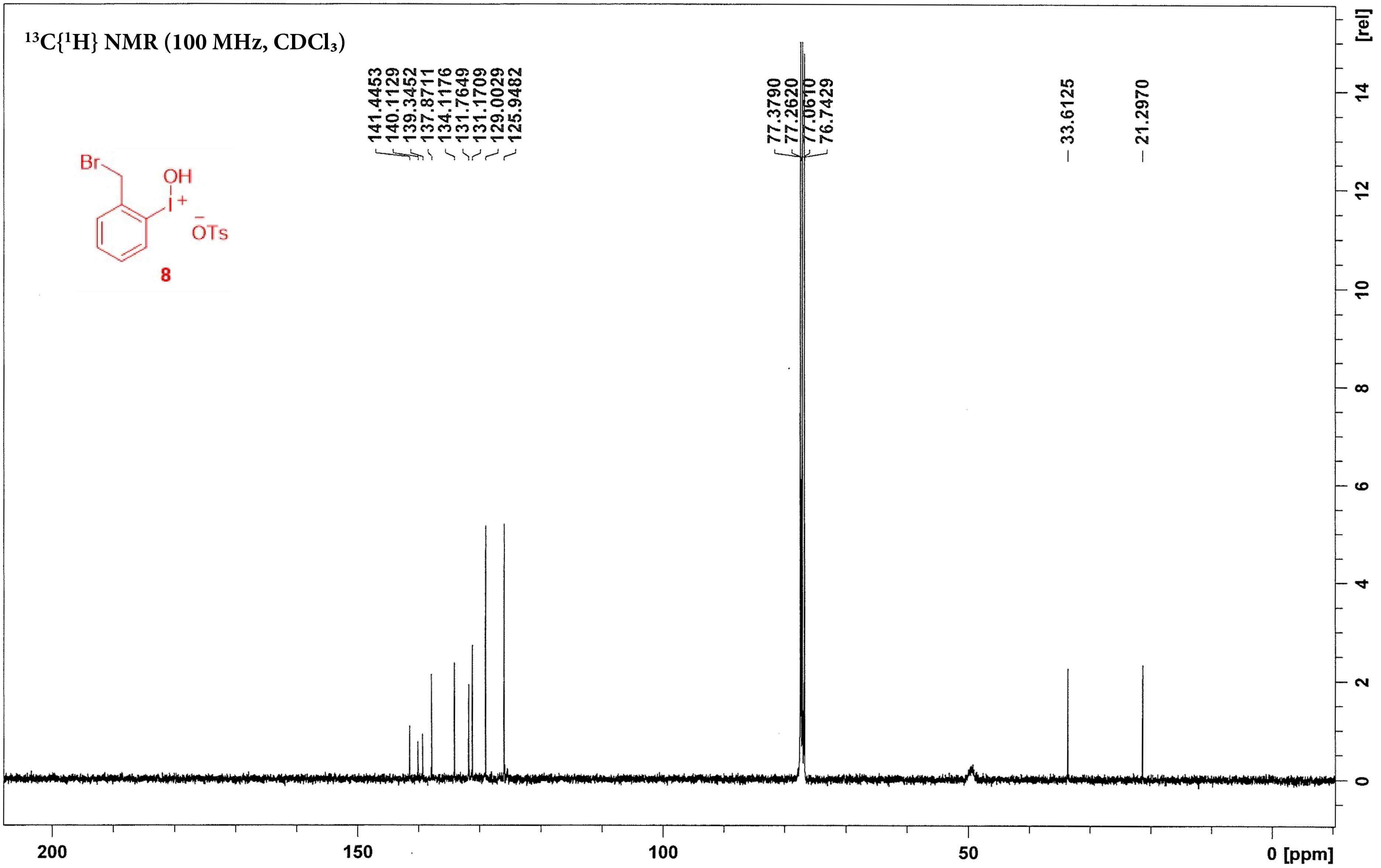

DT-I-Cl-OTs-6-13 1 1 C:\Data\vzgroup

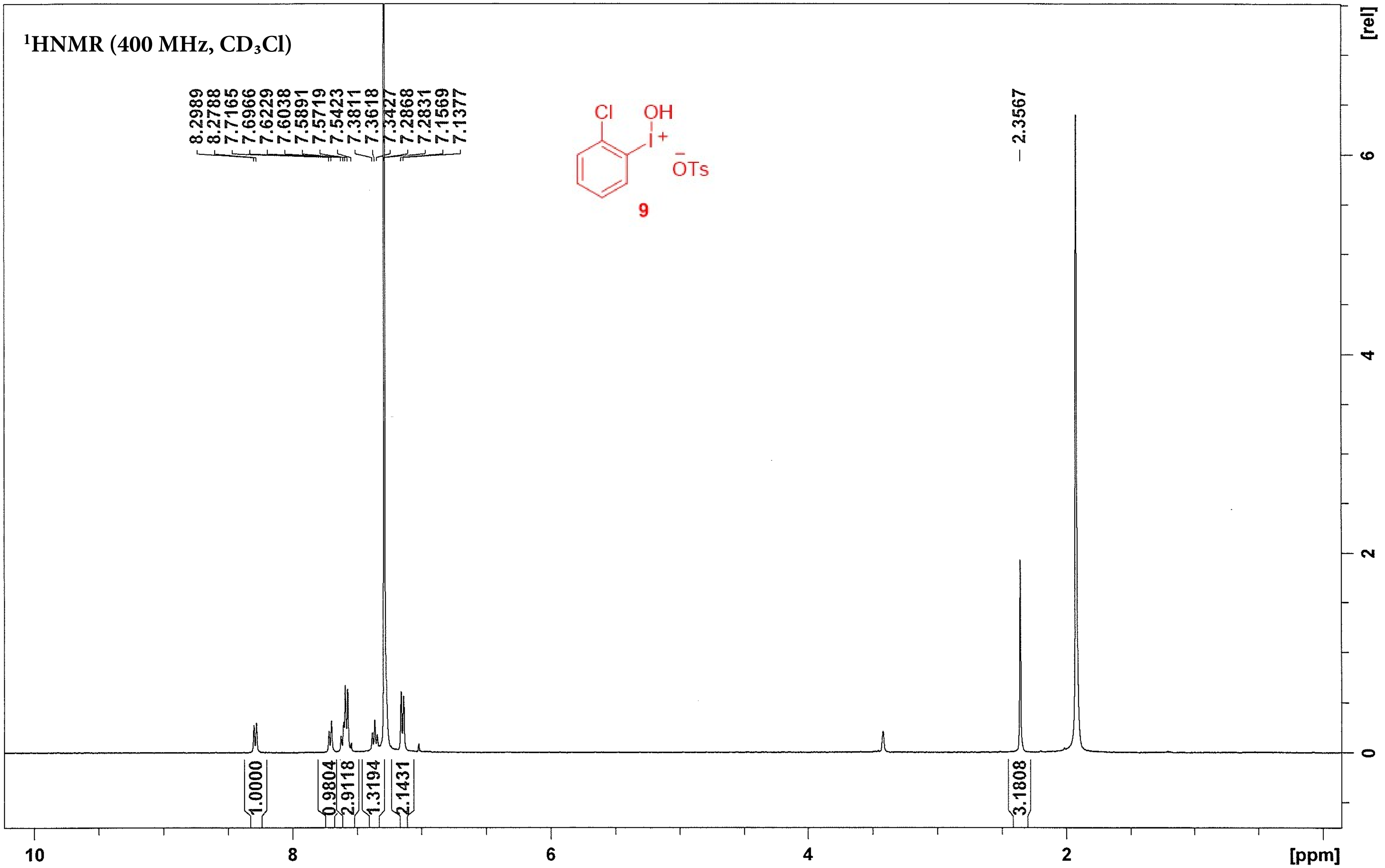

NZ-BH-ClCH3-OTs-1 1 1 D:\nmrdata\vzgroup

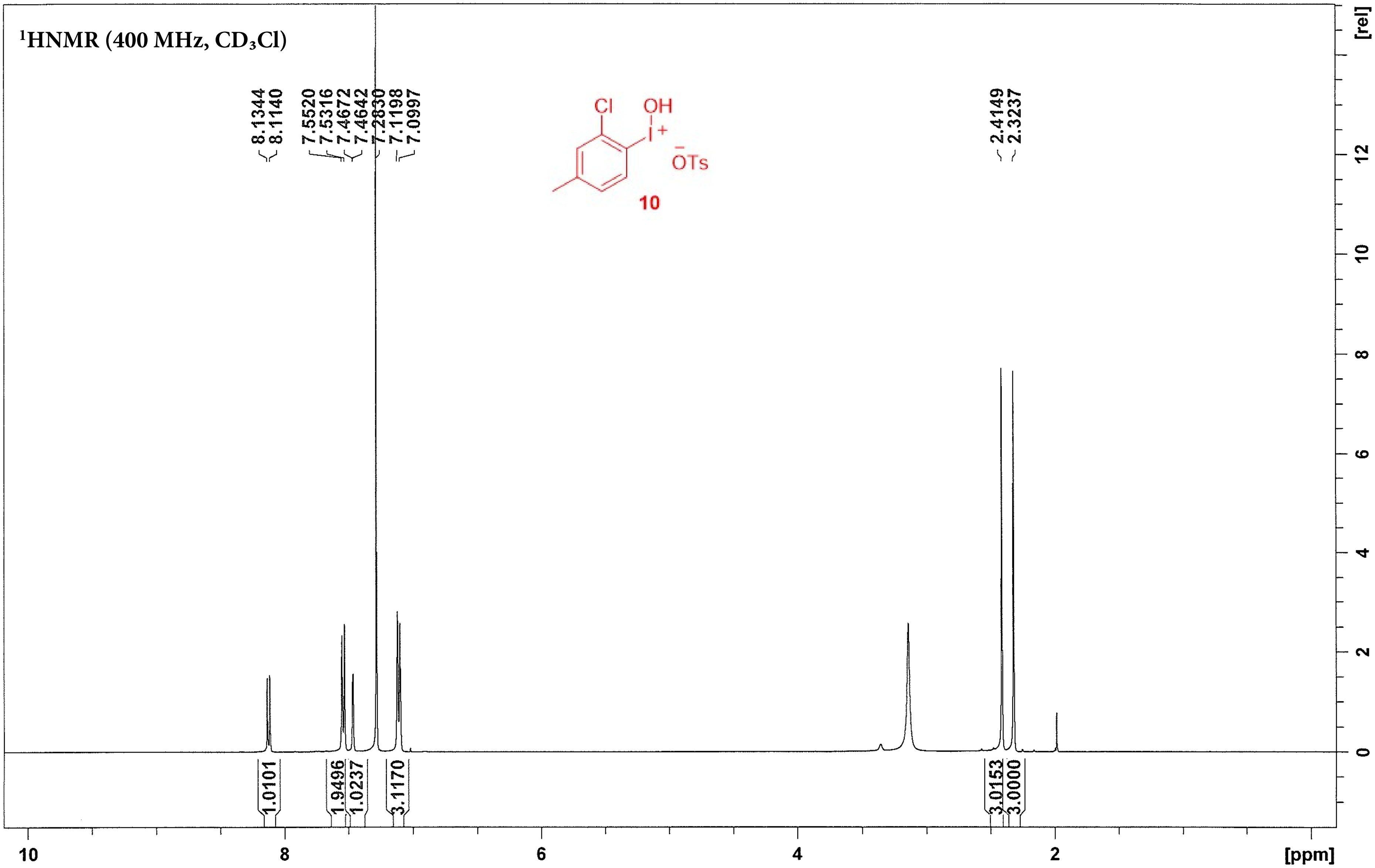

NZ-BH-ClCH3-OTs-1-C 1 1 D:\nmrdata\vzgroup

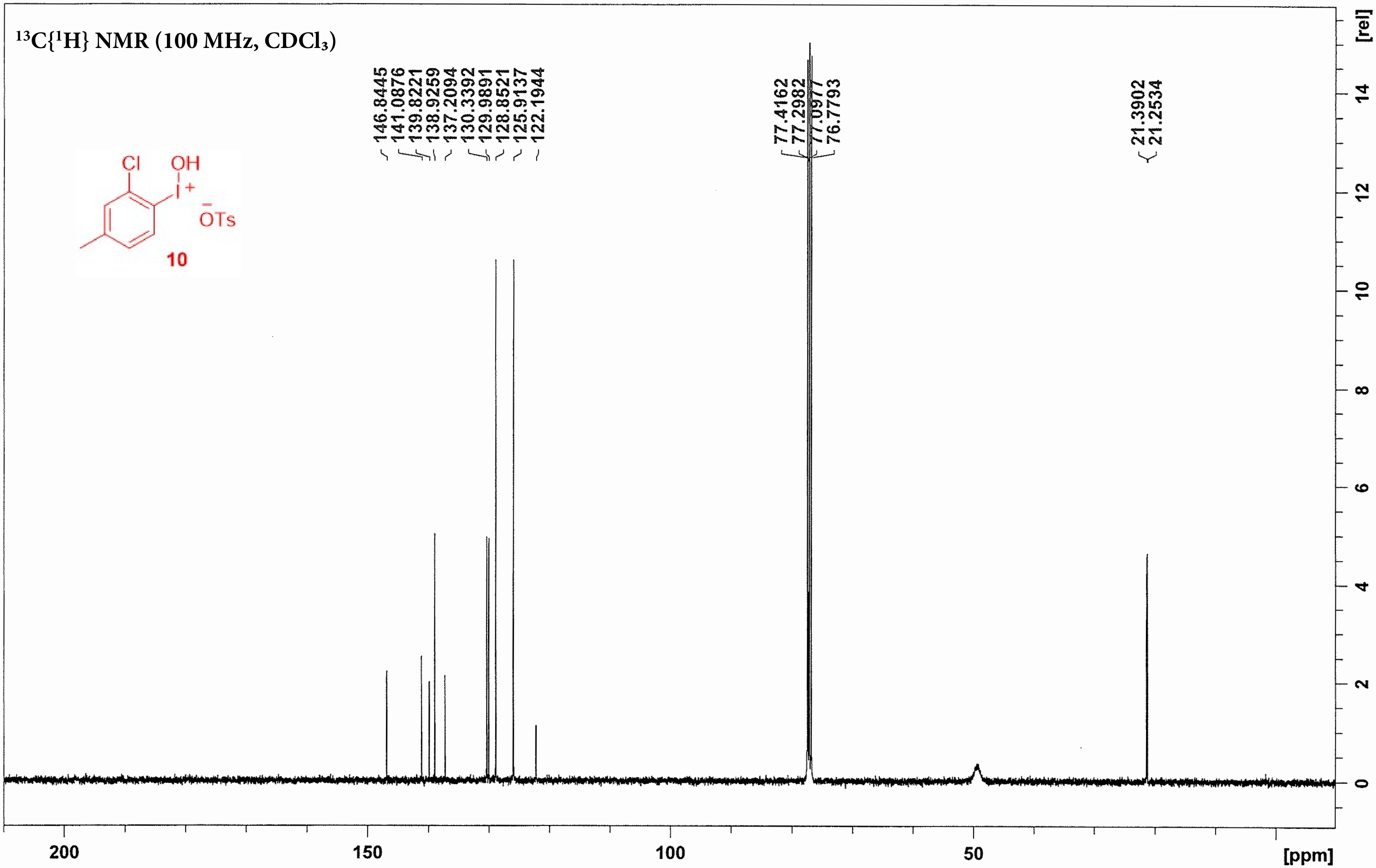

NZ-Br-Mal-MeOH 1 1 D:\nmrdata\vzgroup

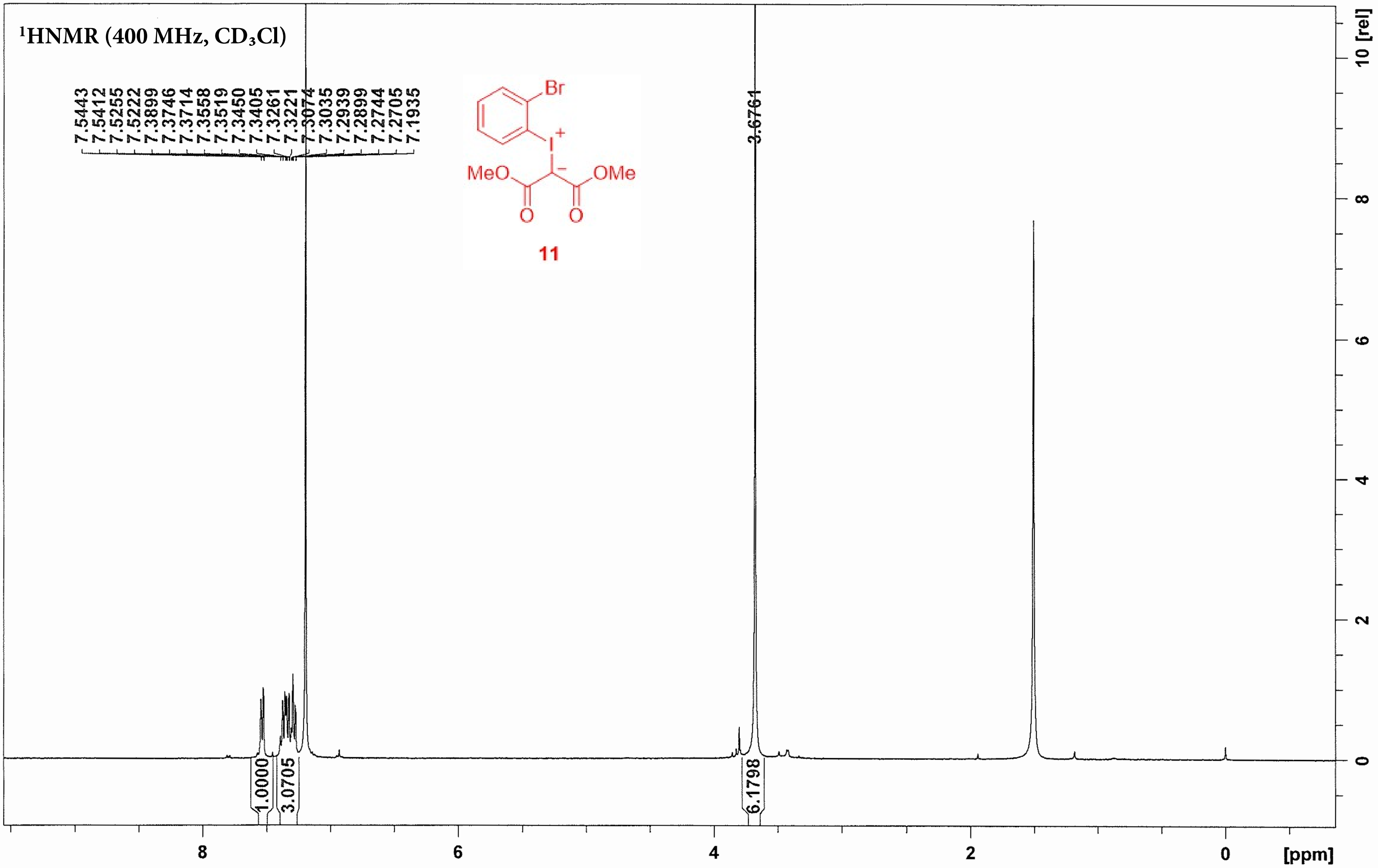

NZ-Br-Mal-C 1 1 D:\nmrdata\vzgroup

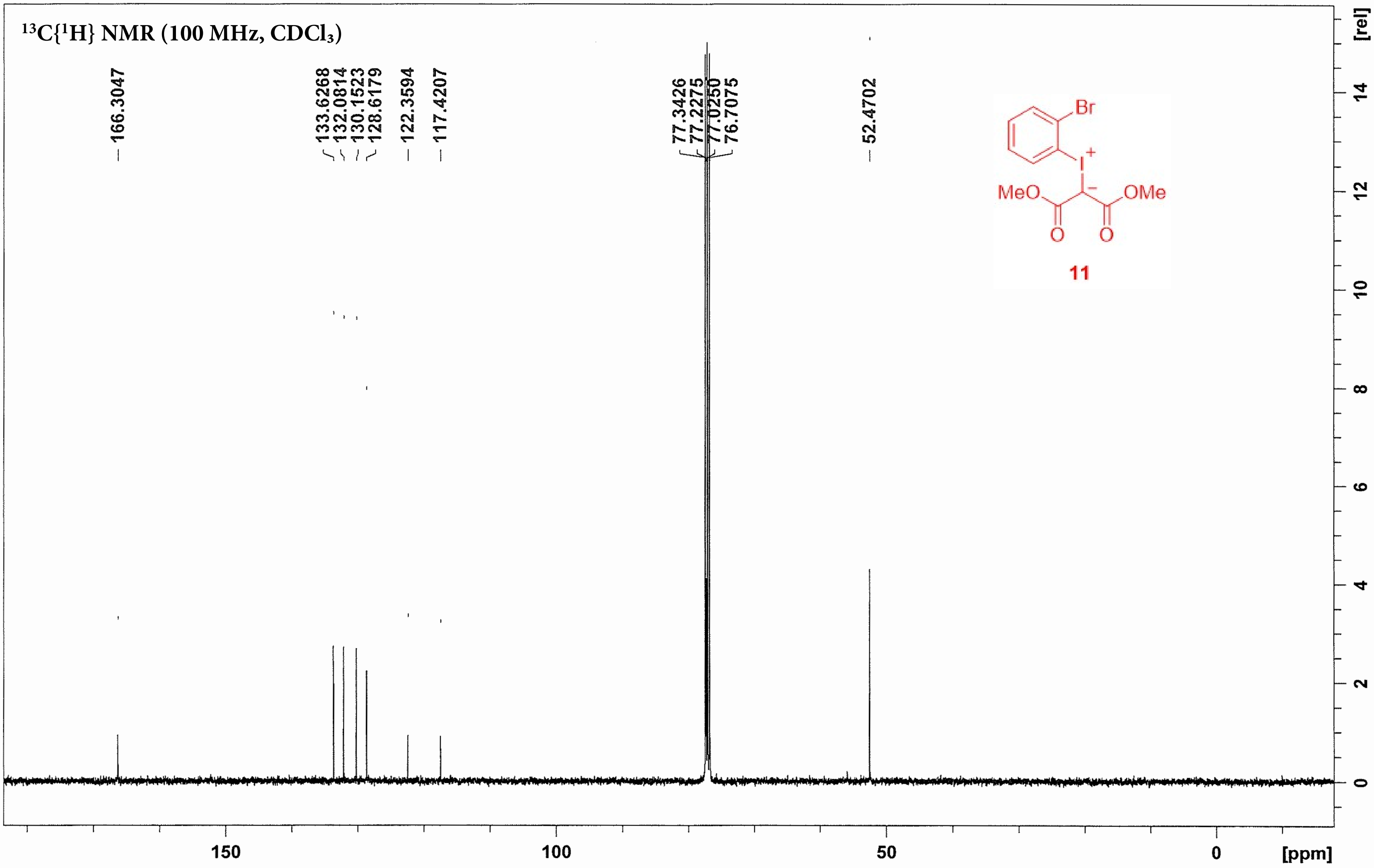

BH-CH3Br-Mal-CH3 1 1 D:\nmrdata\vzgroup

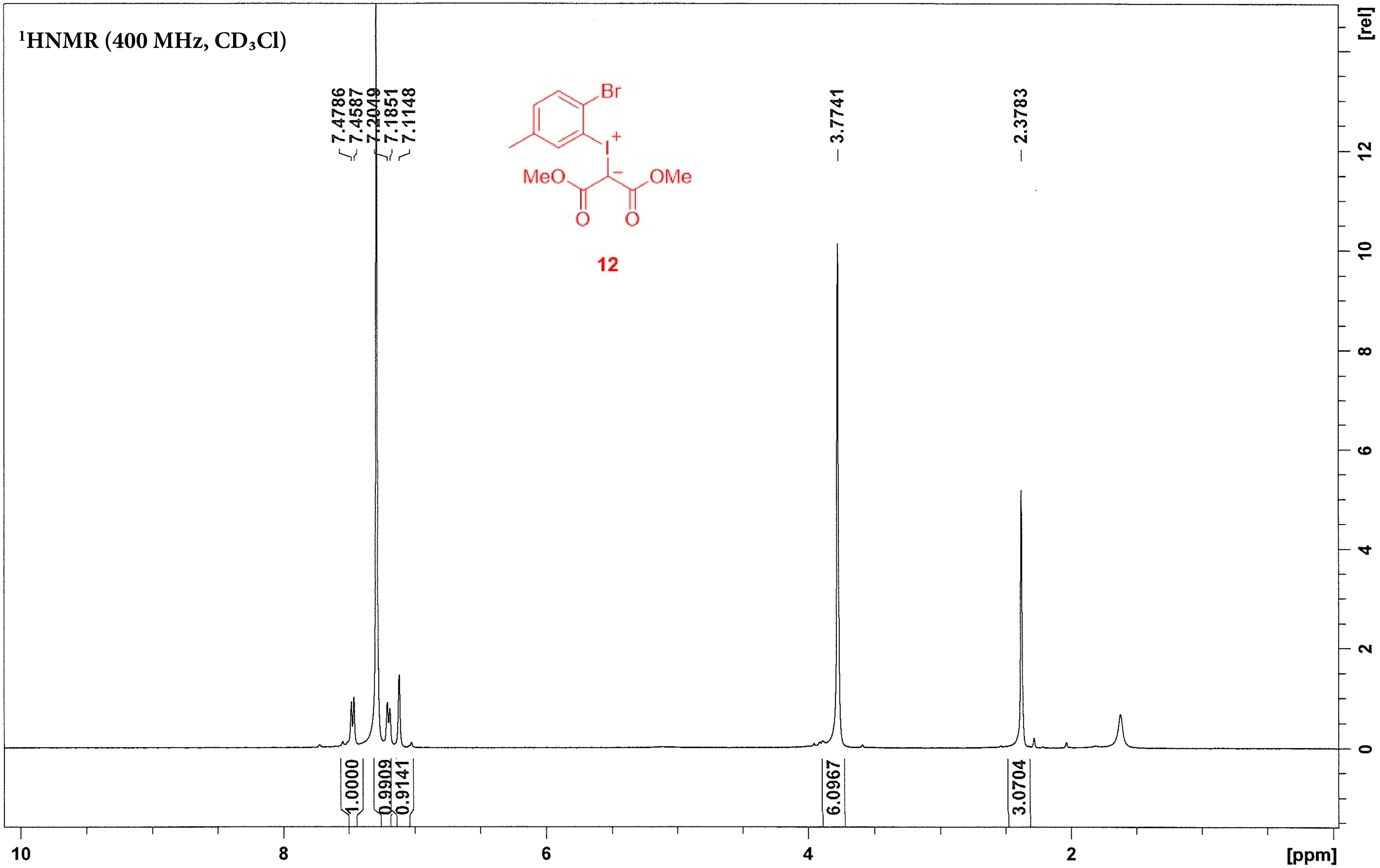

BH-CH3Br-Mal-CH3-C 1 1 D:\nmrdata\vzgroup

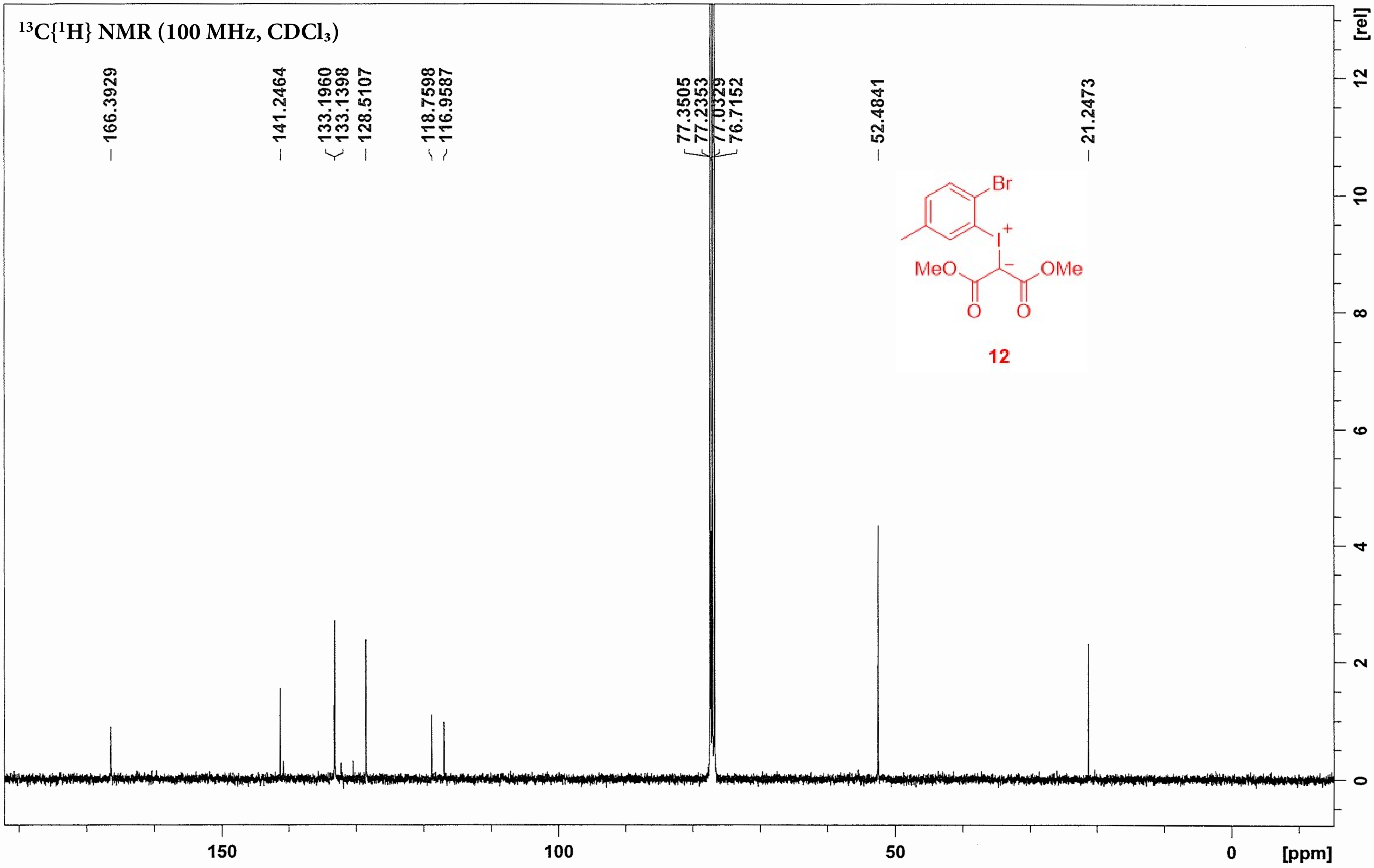

NZ-Cl-Mal-Et2O 1 1 D:\nmrdata\vzgroup

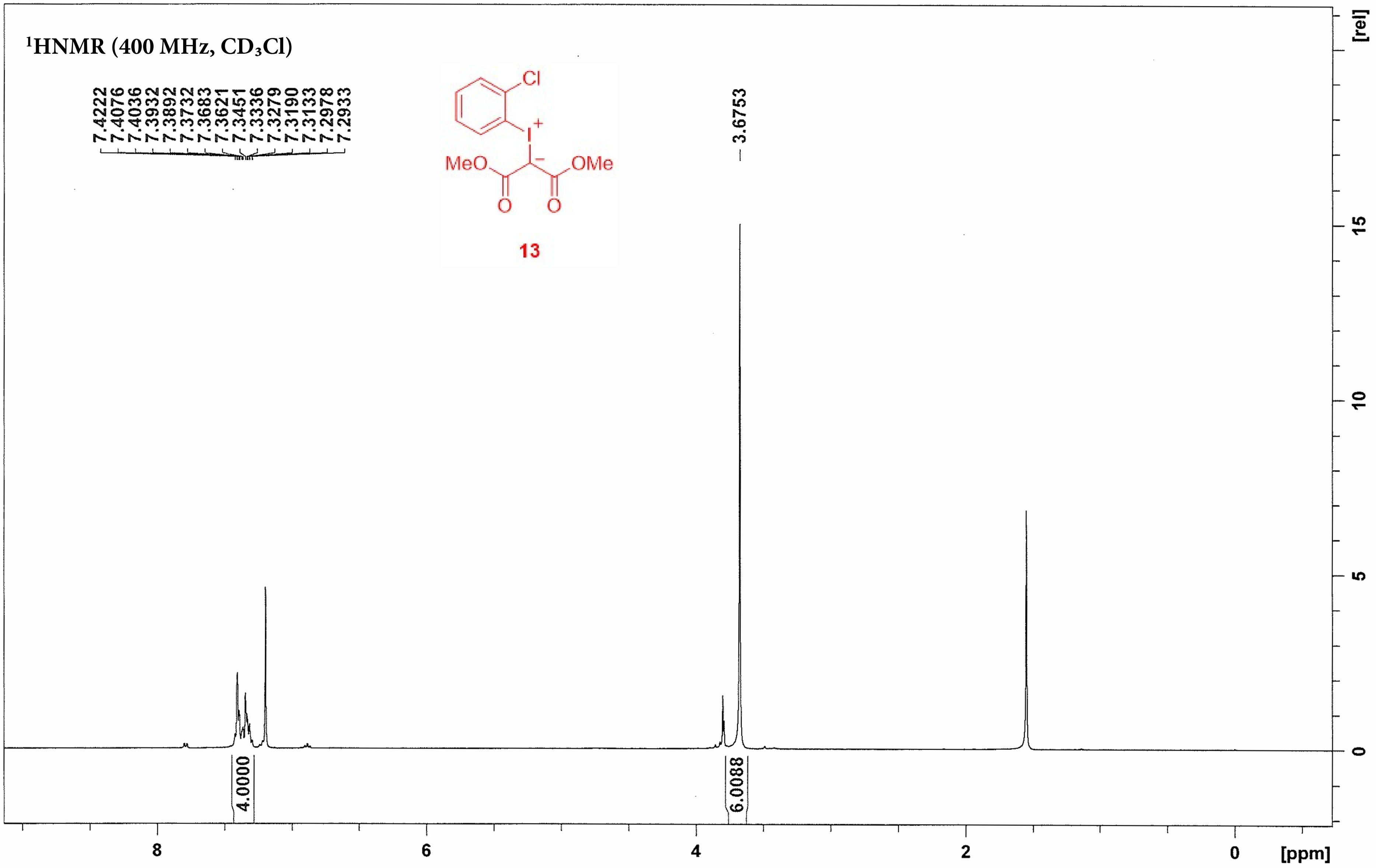

BH-ClCH3-I-Mal-CH3 1 1 D:\nmrdata\vzgroup

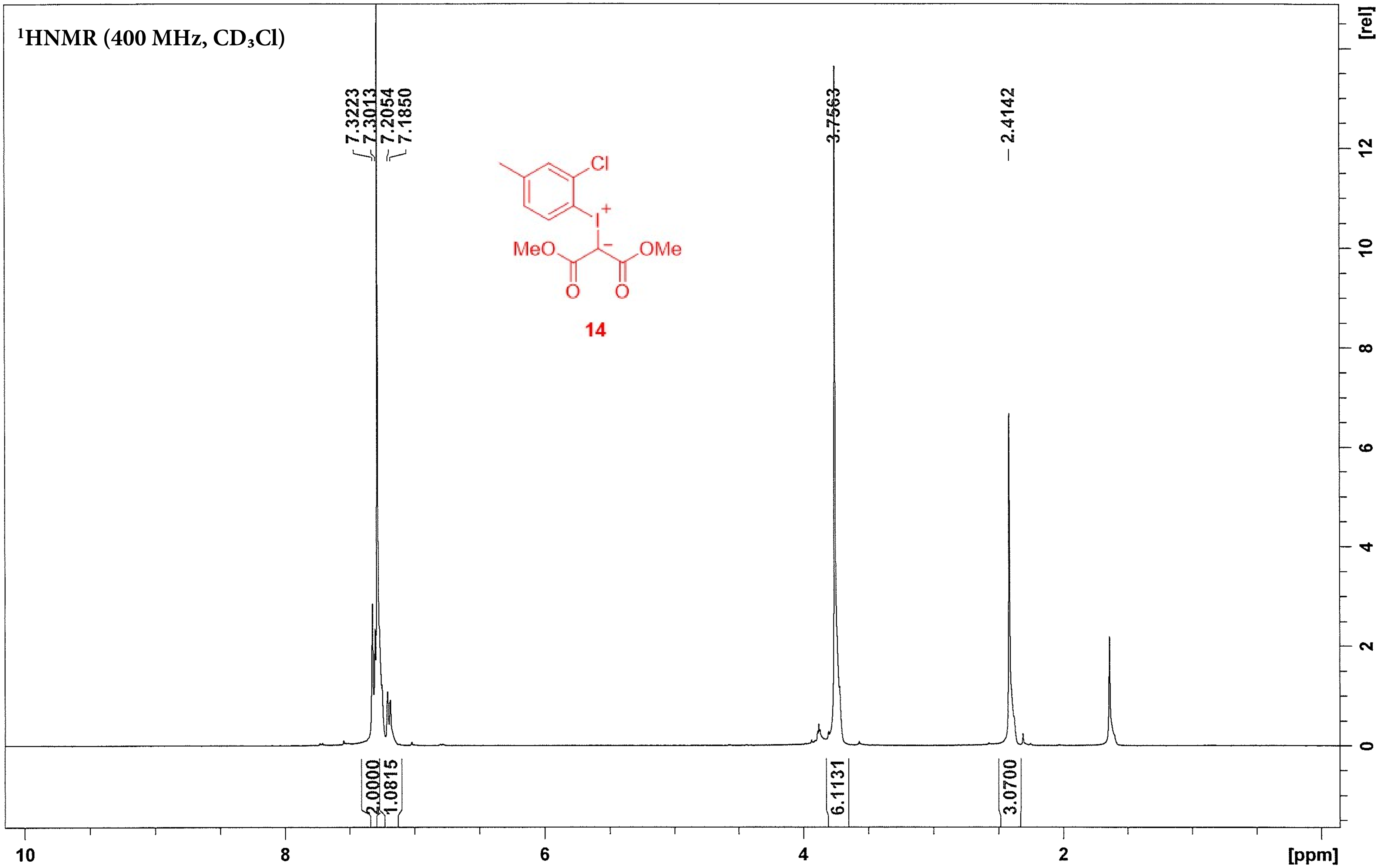

BH-ClCH3-Mal-CH3-C2 1 1 D:\nmrdata\vzgroup

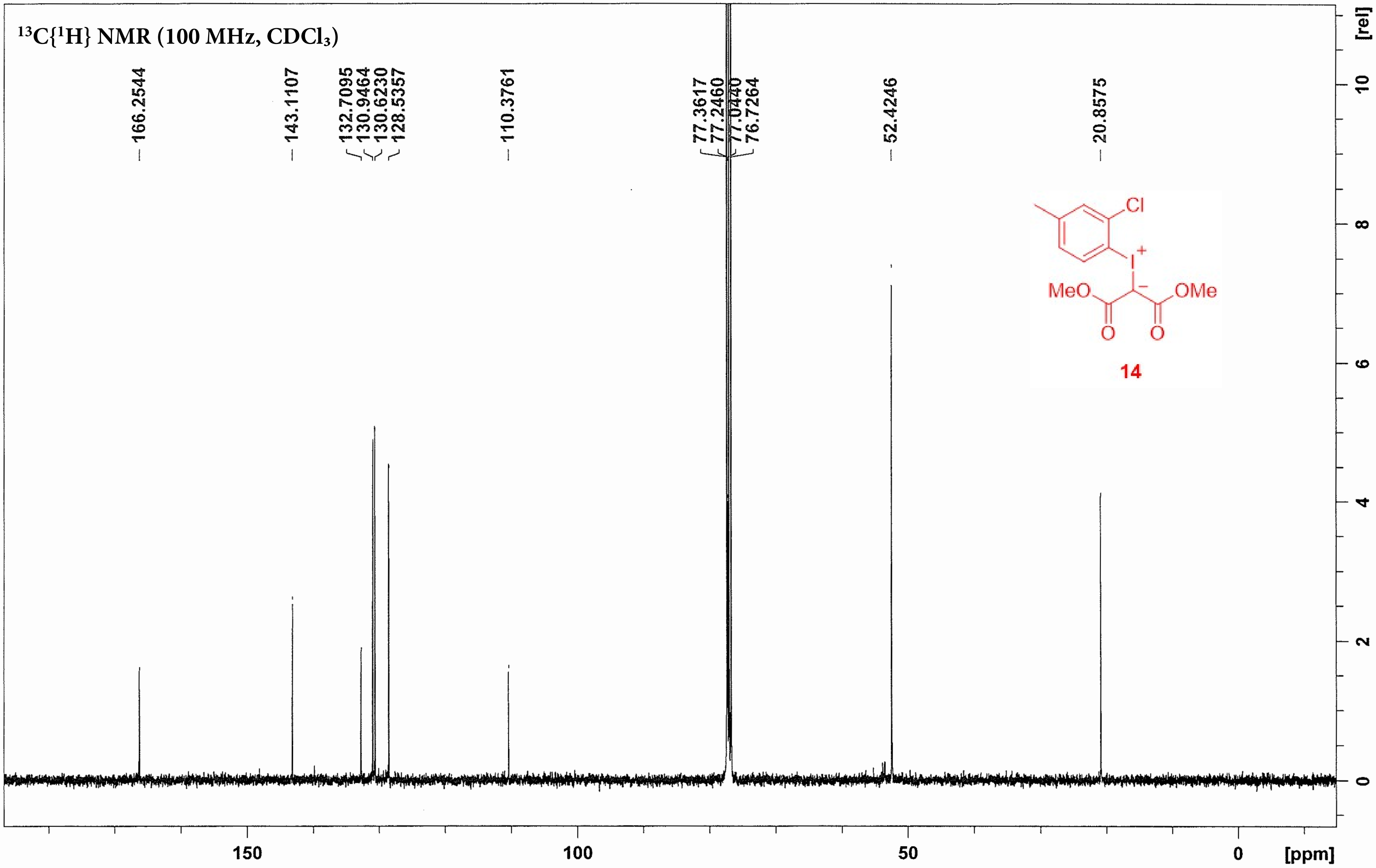

NZ-GH-CH3Br-Mal-Et-Et2O-RT 1 1 D:\nmrdata\vzgroup

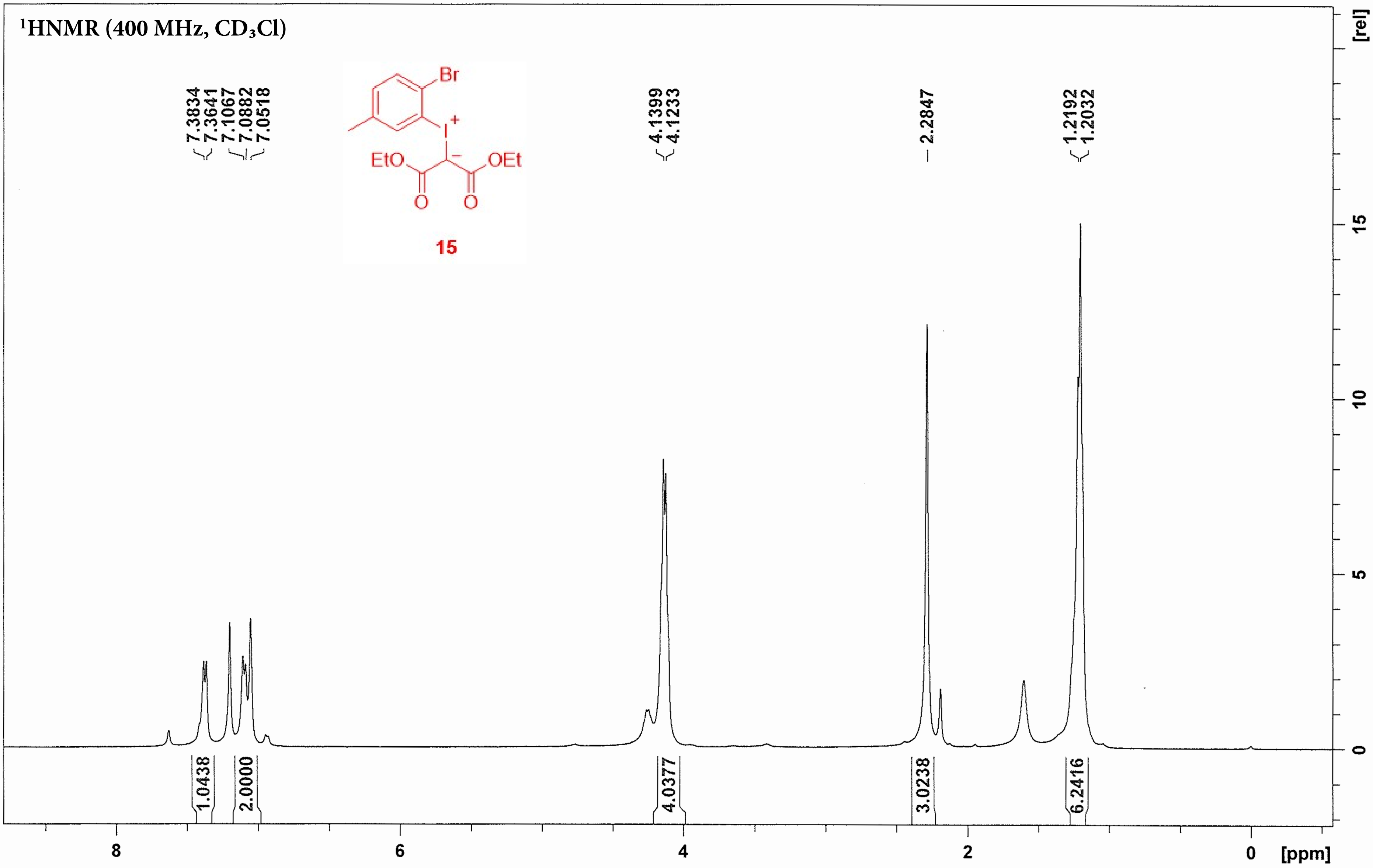

NZ-CH3Br-Mal-Et-C 1 1 D:\nmrdata\vzgroup

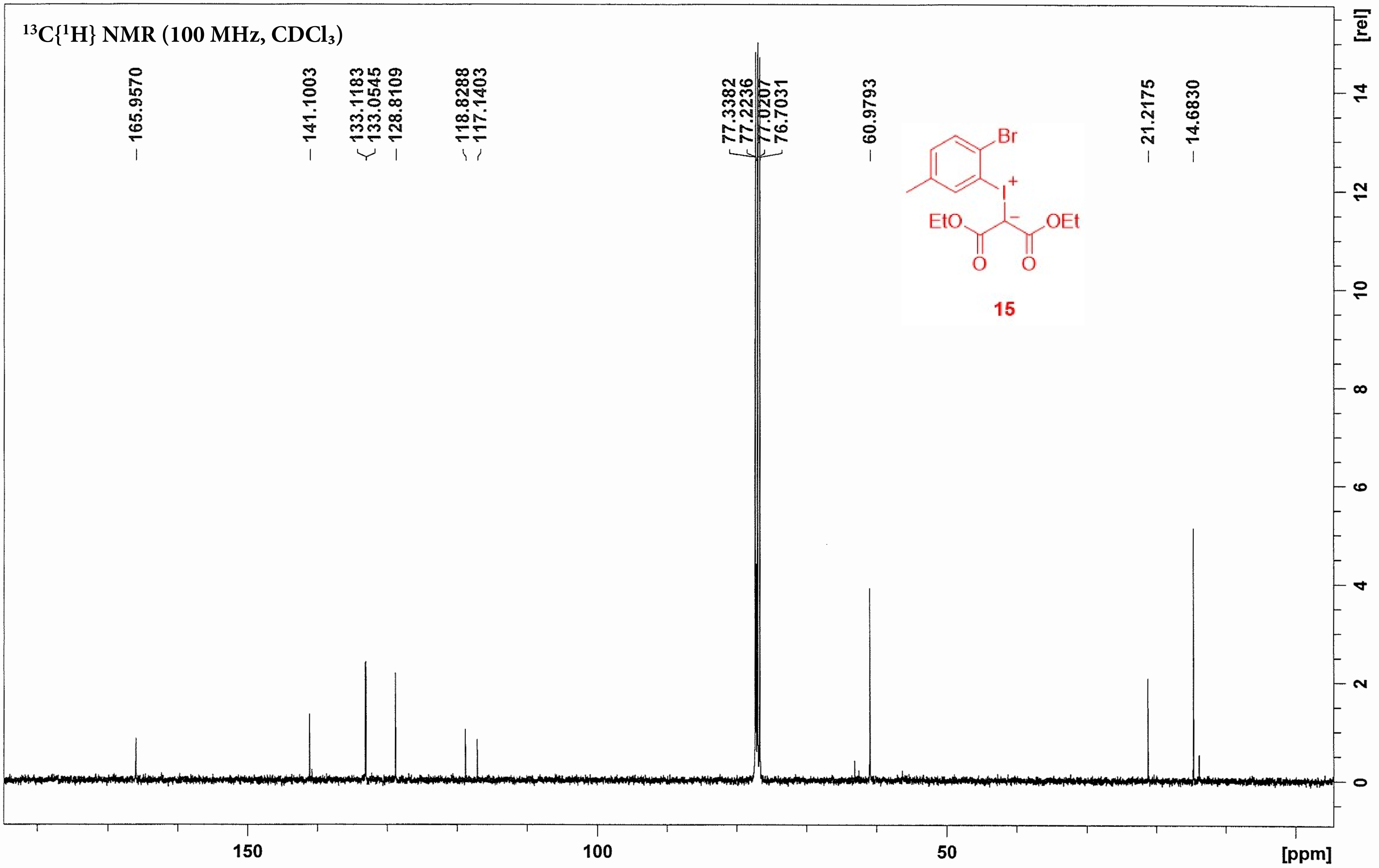

NZ-IosPr-Mal-Br-CH3-H 1 1 D:\nmrdata\vzgroup

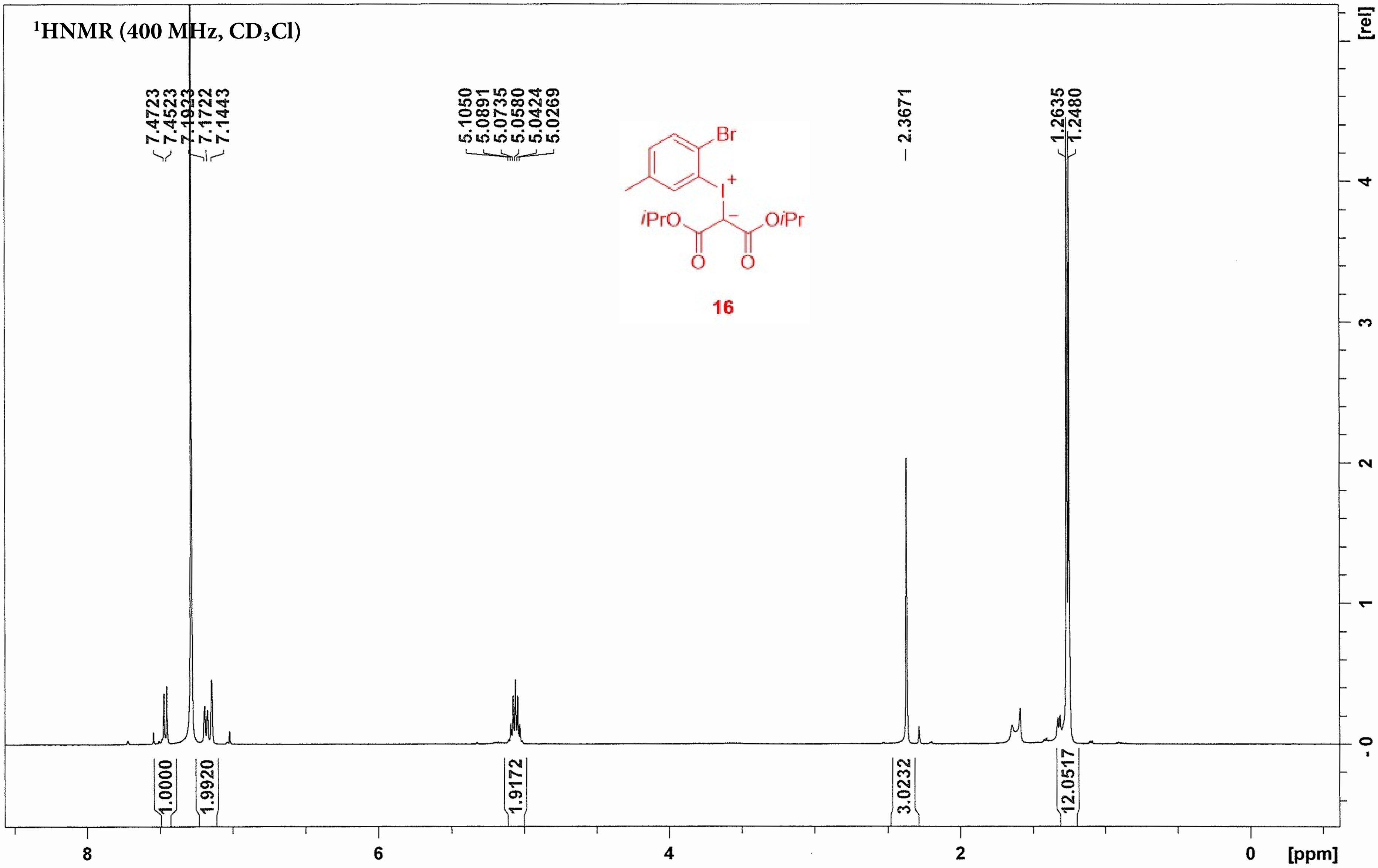

NZ-IsoPr-Mal-CH3Br-C 1 1 D:\nmrdata\vzgroup

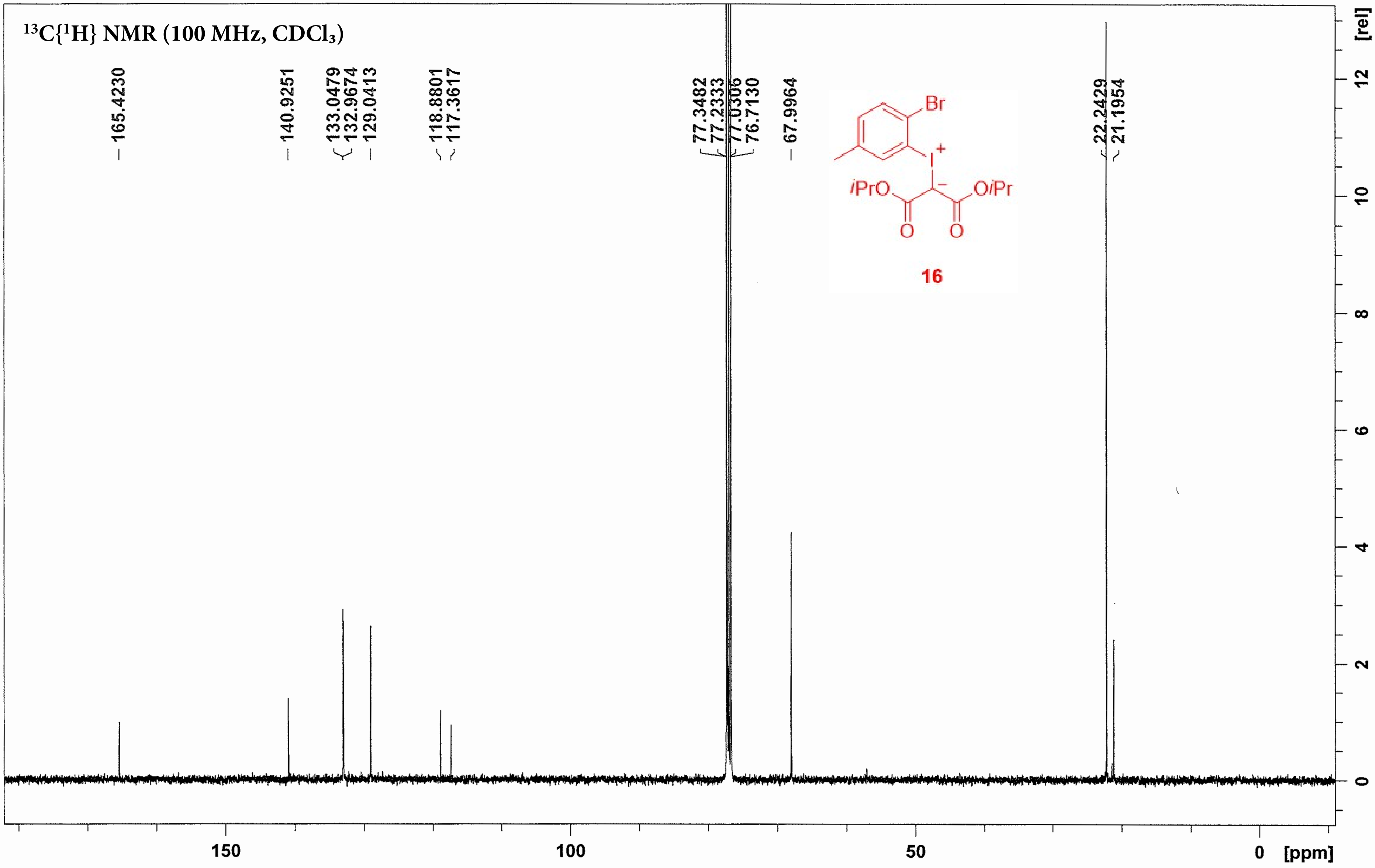

NZ-BrMal-Cy-1317 1 1 D:\nmrdata\vzgroup

^{1}HNMR (400 MHz, CD_3Cl)

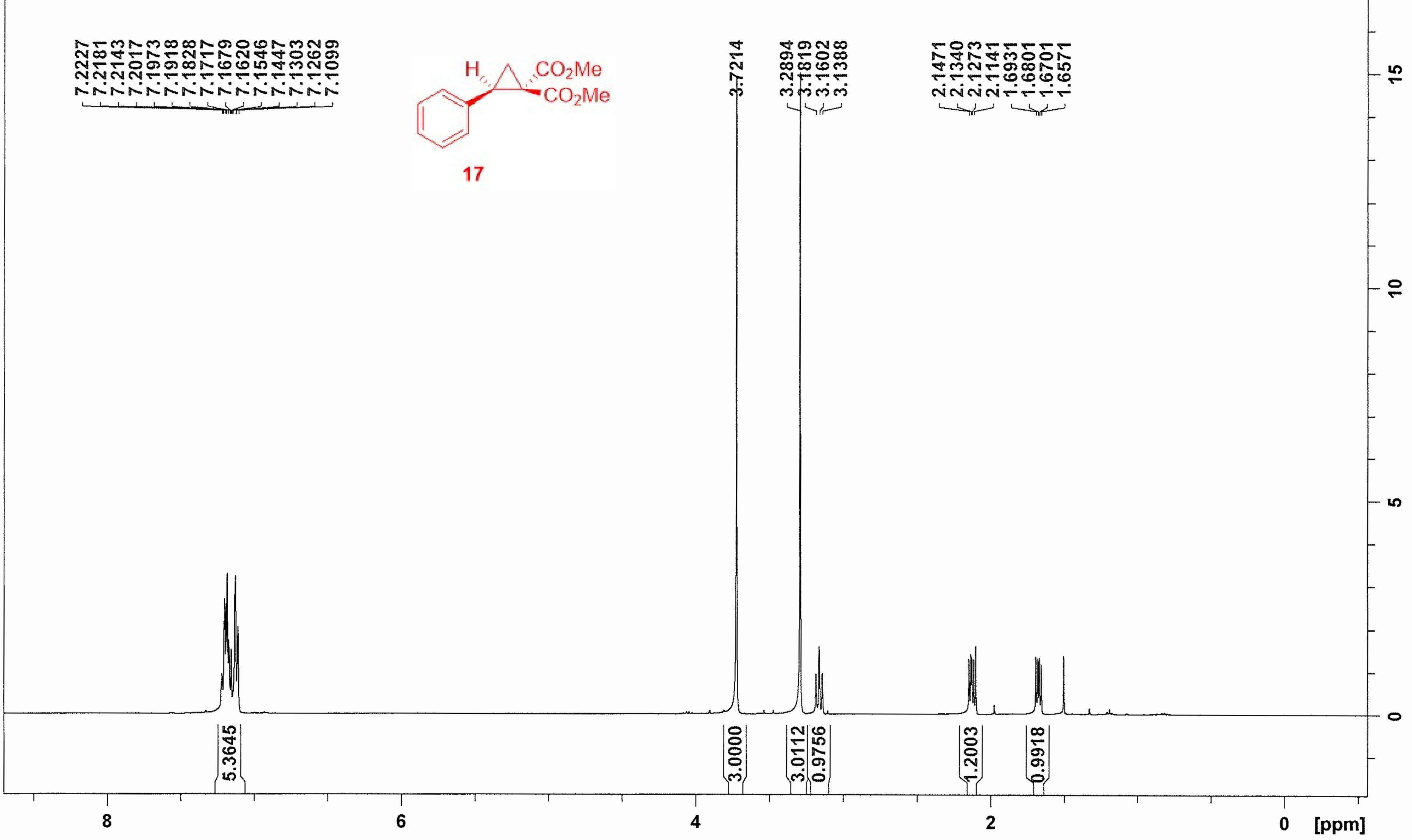

NZ-4-Me-CP-17 1 1 D:\nmrdata\vzgroup

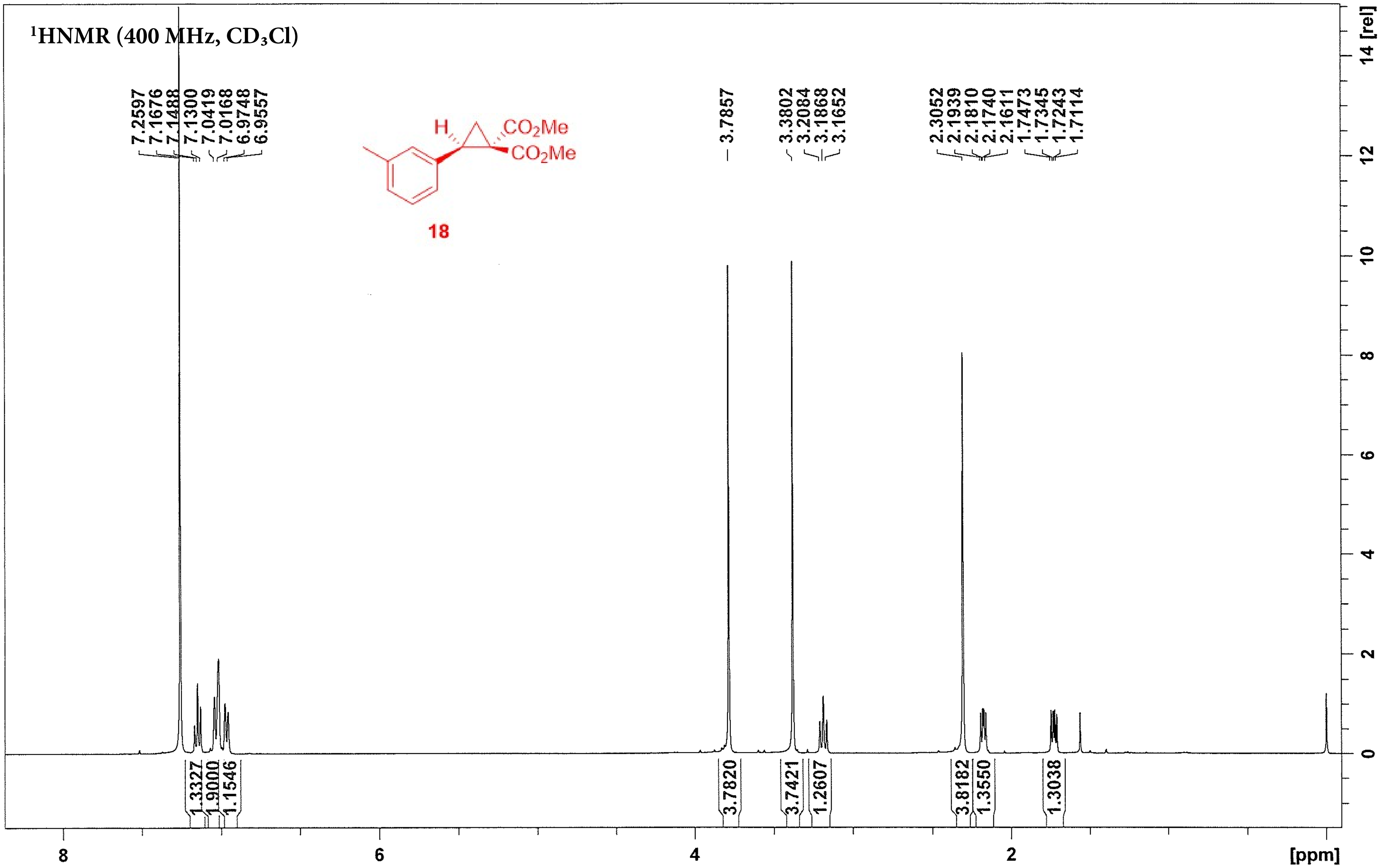

NZ-SK-2Br-CP 1 1 D:\nmrdata\vzgroup

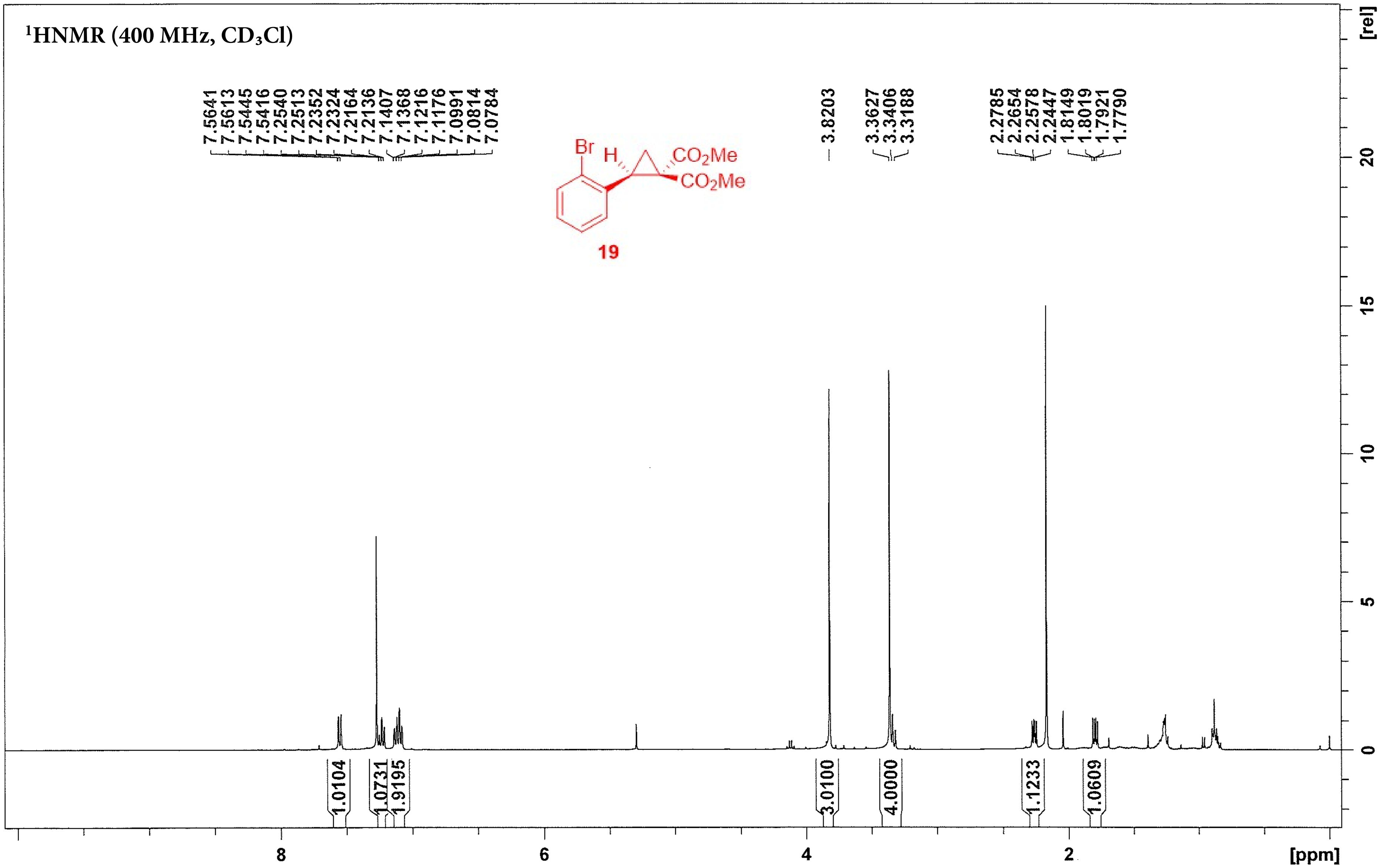

NZ-SK-4-Cl-CP-17 1 1 D:\nmrdata\vzgroup

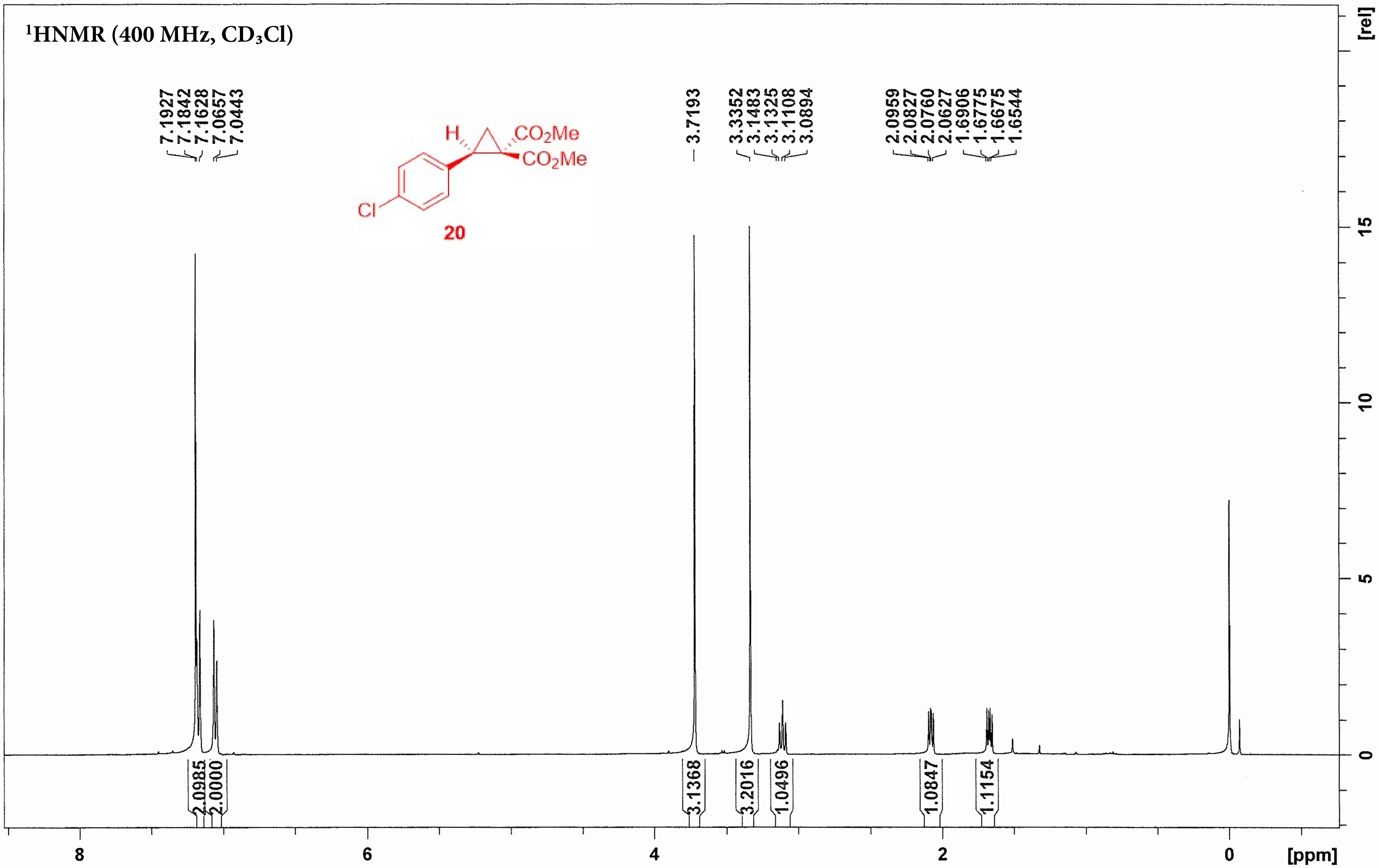

Supplement: Supplementary file 1 [file jo6c00423_si_001.pdf]
